# Supplementary material for: Yorkshire Lung Screening Trial (YLST): protocol for a randomised controlled trial to evaluate invitation to community-based low-dose CT screening for lung cancer versus usual care in a targeted population at risk
Source: BMJ Open. 2020 Sep 10;10(9):e037075. doi: 10.1136/bmjopen-2020-037075 (PMC7485242; doi:10.1136/bmjopen-2020-037075)
Supplement: Author's manuscript [file bmjopen-2020-037075.draft_revisions.pdf]

BMJ Open

BMJ Open is committed to open peer review. As part of this commitment we make the peer review history of every article we publish publicly available.

When an article is published we post the peer reviewers' comments and the authors' responses online. We also post the versions of the paper that were used during peer review. These are the versions that the peer review comments apply to.

The versions of the paper that follow are the versions that were submitted during the peer review process. They are not the versions of record or the final published versions. They should not be cited or distributed as the published version of this manuscript.

BMJ Open is an open access journal and the full, final, typeset and author-corrected version of record of the manuscript is available on our site with no access controls, subscription charges or pay-per-view fees (http://bmjopen.bmj.com).

If you have any questions on BMJ Open's open peer review process please email info.bmjopen@bmj.com

BMJ Open

The Yorkshire Lung Screening Trial (YLST): protocol for a randomised controlled trial to evaluate invitation to community-based low dose computed tomography screening for lung cancer versus usual care in a targeted population at risk.

Journal:	*BMJ Open*
Manuscript ID	bmjopen-2020-037075
Article Type:	Protocol
Date Submitted by the Author:	17-Jan-2020
Complete List of Authors:	Crosbie, Philip; The University of Manchester, Division of Infection, Immunity and Respiratory Medicine<br>Gabe, Rhian; Queen Mary University of London<br>Simmonds, Irene; University of Leeds<br>Kennedy, Martyn; Leeds Teaching Hospitals NHS Trust,<br>Rogerson, Suzanne; Leeds Teaching Hospitals NHS Trust<br>Ahmed, Nazia; University of Leeds<br>Baldwin, David; Nottingham University Hospitals, City Campus<br>Booton, Richard; Manchester University NHS Foundation Trust<br>Cochrane, Ann; The University of York<br>Darby, Michael; Leeds Teaching Hospitals NHS Trust<br>Franks, Kevin; Leeds Teaching Hospitals NHS Trust, Leeds Cancer Centre, St James's University Hospital<br>Hinde, Sebastian; York University, Centre for Health Economics<br>Janes, Sam; University College London, Respiratory Mediine<br>Macleod, Una; Hull York Medical School<br>Messenger, Mike; University of Leeds<br>Moller, Henrik; Kings College London, Thames Cancer Registry<br>Murray, Rachael; The University of Nottingham, Faculty of Medicine & Health Sciences<br>Neal, Richard; University of Leeds,<br>Quaife, Samantha; University College London, Institutional Research Information Servi<br>Sculpher, Mark; University of York,<br>Tharmanathan, Puvanendran; University of York<br>Torgerson, David; The University of York,<br>CALLISTER, Matthew; Leeds Centre for Respiratory Medicine, St James's University Hospital
Keywords:	Respiratory tract tumours < THORACIC MEDICINE, Respiratory tract tumours < ONCOLOGY, Chest imaging < RADIOLOGY & IMAGING

I, the Submitting Author has the right to grant and does grant on behalf of all authors of the Work (as defined in the below author licence), an exclusive licence and/or a non-exclusive licence for contributions from authors who are: i) UK Crown employees; ii) where BMJ has agreed a CC-BY licence shall apply, and/or iii) in accordance with the terms applicable for US Federal Government officers or employees acting as part of their official duties; on a worldwide, perpetual, irrevocable, royalty-free basis to BMJ Publishing Group Ltd ("BMJ") its licensees and where the relevant Journal is co-owned by BMJ to the co-owners of the Journal, to publish the Work in this journal and any other BMJ products and to exploit all rights, as set out in our licence.

The Submitting Author accepts and understands that any supply made under these terms is made by BMJ to the Submitting Author unless you are acting as an employee on behalf of your employer or a postgraduate student of an affiliated institution which is paying any applicable article publishing charge ("APC") for Open Access articles. Where the Submitting Author wishes to make the Work available on an Open Access basis (and intends to pay the relevant APC), the terms of reuse of such Open Access shall be governed by a Creative Commons licence – details of these licences and which Creative Commons licence will apply to this Work are set out in our licence referred to above.

Other than as permitted in any relevant BMJ Author's Self Archiving Policies, I confirm this Work has not been accepted for publication elsewhere, is not being considered for publication elsewhere and does not duplicate material already published. I confirm all authors consent to publication of this Work and authorise the granting of this licence.

For peer review only

The Yorkshire Lung Screening Trial (YLST): protocol for a randomised controlled trial to evaluate invitation to community-based low dose computed tomography screening for lung cancer versus usual care in a targeted population at risk.

Philip AJ Crosbie[1,2], Rhian Gabe[3], Irene Simmonds[4], Martyn Kennedy[5], Suzanne Rogerson[5], Nazia Ahmed[4], David Baldwin[6], Richard Booton[1,2,7], Ann Cochrane[8], Mike Darby[5], Kevin Franks[5], Sebastian Hinde[9], Sam M Janes[10], Una MacLeod[11], Mike Messenger[4], Henrik Møller[12], Rachael L Murray[13], Richard D Neal[4], Samantha L Quaife[14], Mark Sculpher[9], Puvan Tharmanathan[8], David Torgerson[8], Matthew E J Callister[5]*.

[1]Division of Infection, Immunity & Respiratory Medicine, University of Manchester, UK. [2]Manchester Thoracic Oncology Centre, Manchester University NHS Foundation Trust, Manchester, UK. [3]Centre for Cancer Prevention, Queen Mary University of London, UK. [4]Institute of Health Sciences, University of Leeds, UK. [5]Leeds Teaching Hospitals NHS Trust, Leeds, UK. [6]Department of Respiratory Medicine, Nottingham University Hospitals, Nottingham, UK. [7]Manchester Academic Health Science Centre, University of Manchester, UK. [8]York Trials Unit, York, UK. [9]Centre for Health Economics, University of York, UK. [10]Lungs for Living Research Centre, UCL Respiratory, University College London, UK. [11]Hull York Medical School, University of Hull, UK. [12]King's College London, London, UK. [13]Division of Epidemiology and Public Health, University of Nottingham, UK. [14]Research Department of Behavioural Science and Health, University College London, UK.

***Corresponding author:** Dr Mat Callister, St James's University Hospital, Beckett Street, Leeds, UK. LS9 7TF. Tel +44 (0) 113 206 4159. Email: matthew.callister@nhs.net

Word count: 4,718

International Standard Registered Clinical Trial Number: ISRCTN42704678

Keywords: lung cancer screening, lung health check, community, lung cancer risk

For peer review only - http://bmjopen.bmj.com/site/about/guidelines.xhtml

Abstract

Introduction: Lung cancer is the world's leading cause of cancer death. Low dose computed tomography (LDCT) screening reduced lung cancer mortality by 20% in the US National Lung Screening Trial (NLST). Here we present the Yorkshire Lung Screening Trial (YLST) which will address key questions of relevance for screening implementation.

Methods and analysis: Using a single-consent Zelen's design, ever-smokers aged 55 to 80 years registered with a GP in Leeds will be randomised (1:1) to invitation to a telephone-based risk-assessment for a Lung Health Check (LHC) or to usual care. The anticipated number randomised by household is 62,980 individuals. Responders at high risk will be invited for LDCT scanning for lung cancer on a mobile van in the community. There will be 2 rounds of screening at an interval of 2 years. Primary objectives are (i) measure participation rates, (ii) compare the performance of $PLCO_{M2012}$ (threshold ≥1.51%), LLP_{v2} (threshold ≥5%) and US Preventive Services Task Force (USPSTF) eligibility criteria for screening population selection, and (iii) assess lung cancer outcomes in the intervention and usual care arms. Secondary evaluations include health economics, quality of life, smoking rates according to intervention arm, screening programme performance with ancillary biomarker, and smoking cessation studies.

Ethics and dissemination: The study has been approved by Greater Manchester West research ethics committee (18-NW-0012) and the Health Research Authority following review by the Confidentiality Advisory Group (CAG). Results will be disseminated through publication in peer-reviewed scientific journals, presentation at conferences and on the YLST website.

For peer review only - http://bmjopen.bmj.com/site/about/guidelines.xhtml

Strengths and limitations of this study

- Prospective evaluation of three criteria for screening population selection to inform the optimal approach for screening implementation.
- The Zelen's study design and unscreened control group (current standard of care in the UK) will allow us to assess the impact of lung cancer screening at a population level.
- Prospective evaluation of participation rates and clinical outcomes of community-based lung cancer screening with biennial low dose CT scans.
- A nested sub-study within YLST (the Yorkshire Enhanced Stop Smoking (YESS) Trial) will evaluate the effectiveness of a fully integrated co-located stop smoking service in a screening programme.
- The study is not powered to assess the impact of screening on lung cancer specific mortality.

For peer review only

For peer review only - http://bmjopen.bmj.com/site/about/guidelines.xhtml

Introduction

Lung cancer is the leading cause of cancer mortality nationwide, responsible for approximately 30,000 deaths / year in England and Wales. Low dose computed tomography (LDCT) screening reduced mortality in the US National Lung Screening Trial (NLST) which randomised 53,439 participants at high risk to annual LDCT or chest x-ray (CXR) screening for two years (1). LDCT screening reduced lung cancer specific and all-cause mortality by 20% and 6.7% respectively. This finding has recently been confirmed by NELSON trial investigators (2), although formal publication of results is awaited. The MILD study demonstrated a 39% reduction in lung cancer specific mortality over 10 years with LDCT screening (3). In 2014, the US Preventive Services Task Force (USPSTF) recommended LDCT screening for lung cancer based on the NLST findings, extending the upper age to 80 years (4, 5). Despite on-going implementation in the US, several important issues remain unresolved, including optimising identification of high-risk individuals for screening, embedding smoking-cessation in screening programmes, and improving uptake in those at highest risk (6).

Screening selection criteria

Within NLST, very few deaths were prevented in the two lowest lung cancer risk quintiles (7). In addition only 27% of patients diagnosed with lung cancer in the US (8) and 35% in Yorkshire would have been eligible for screening by NLST criteria (9). Composite risk prediction tools show improved sensitivity, specificity and positive predictive value when retrospectively compared to NLST criteria (10). Such tools may therefore identify a greater number of people with lung cancer, and so improve the effectiveness of screening. However, they also tend to identify an older cohort, and thus some of the effect on life years gained and therefore cost-effectiveness may be attenuated (11).

For peer review only - http://bmjopen.bmj.com/site/about/guidelines.xhtml

The Yorkshire Lung Screening Trial (YLST) will prospectively assess three proposed methods of screening population selection: USPSTF, Liverpool Lung Project (LLP), and Prostate, Lung, Colorectal and Ovarian (PLCO) Cancer Screening Trial models. NLST eligibility criteria were age 55-74, ≥30 pack years and smoked within 15 years (1). The USPSTF concluded that screening should be extended to age 80 (12) and issued a recommendation in 2014 (4, 5). The $PLCO_{M2012}$ risk prediction model was derived in 80,375 participants of the PLCO study, and validated in a separate cohort of 37,332 (13). Lung cancer risk ≥1.51% over 6 years was identified as the threshold defining consistent reduced mortality with LDCT screening (10). The UK Lung Cancer Pilot Screening Trial (UKLS) used the LLP model set at a threshold of ≥5% over 5 years to select participants for screening (14). YLST will prospectively assess the performance of USPSTF criteria, the $PLCO_{M2012}$ risk prediction model (6-year risk threshold ≥1.51%) and LLP (version 2) model (5-year risk threshold ≥5%).

Optimising participation for those at most risk

Lung cancer risk is highest within lower socio-economic status (SES) communities (15). Both low SES and current smoking are associated with lower participation in cancer screening programmes (16, 17) and research studies (18-20). In UKLS transport difficulties were the most commonly reported practical barrier to participation (21) especially in lower socioeconomic quintiles, while emotional barriers including higher affective risk perceptions were more frequently reported by smokers. Attendees of Manchester's LHC pilot expressed a preference for community-based screening (22). A randomised trial of mobile mammography for breast screening showed increased participation rates especially in older and low income populations (23). Interventions shown to improve participation in colorectal screening include a primary care endorsement letter, advanced notification of the screening offer and reminder re-invitations (24-28). Enhanced participant information leaflets as well as invitations targeted to address psychological barriers are also promising (29), and in the Lung Screen Uptake Trial

For peer review only - http://bmjopen.bmj.com/site/about/guidelines.xhtml

a low information burden, targeted and stepped invitation approach improved uptake in the lowest SES quintile (30). These factors form part of our invitational strategy.

Cost effectiveness of LDCT screening

LDCT screening needs to ensure the considerable financial investment results in the maximum healthcare gain. Previous UK studies have estimated the incremental cost-effectiveness ratio (ICER) of screening as £8,466 per quality adjusted life year (QALY) in the UKLS (14), £10,069/QALY for mobile LDCT screening in Manchester (31) and £28,169/QALY in a systematic review based analysis (32). The resultant variation in conclusions regarding the cost-effectiveness of screening, driven by different model approaches and populations, highlights the need for further research. By assessing screening yield using different risk tools and thresholds, it will be possible to compare both the clinical and cost effectiveness of different selection criteria.

Rationale

Questions remain about whether the mortality reduction demonstrated in NLST can be translated into routine clinical care. Implementation studies are required to demonstrate participation amongst people at highest risk of lung cancer particularly from deprived populations where the burden of disease is greatest. Basing screening in convenient community locations is one proposed strategy to increase uptake, especially in deprived populations (22). Invitation to a "Lung Health Check" (LHC) not "lung cancer screening" is an approach that has been successfully used to increase participation in previous UK studies leading to screening of higher risk individuals (33-35). Furthermore, clarification of the optimal strategy for defining a high-risk population for screening in the UK would aid any subsequent roll-out of a national screening programme.

For peer review only - http://bmjopen.bmj.com/site/about/guidelines.xhtml

Objectives

The primary objectives of the study are:

1. To measure participation rates of a community-based lung cancer screening programme.
2. To compare the performance of USPSTF, $PLCO_{M2012}$ (≥1.51%) and LLP_{v2} (≥5%) criteria for identifying individuals at high risk of lung cancer for LDCT screening.
3. To assess the clinical outcomes of invitation to targeted community based LDCT screening for lung cancer versus usual care (no invitation).

The study's secondary objectives are:

1. To undertake a health economic evaluation of community based LDCT screening for lung cancer, specifically comparing the health economic impact of the three selection criteria (USPSTF, PLCO, LLP).
2. To evaluate the performance of the screening programme.
3. To determine the effect of invitation to a screening programme on smoking rates comparing intervention and usual care arms.

Outcome measures

The primary outcome measures are:

- The proportion of the study population allocated to intervention who undergo telephone assessment and are screened according to the USPSTF, PLCO and LLP criteria.
- Lung cancers identified in participants selected for LDCT screening according to the USPSTF, PLCO and LLP criteria over two rounds of biennial screening.
- The incidence of advanced lung cancer in the intervention and control arms over the course of the study.

For peer review only - http://bmjopen.bmj.com/site/about/guidelines.xhtml

The secondary outcome measures are:

- Incremental cost-effectiveness ratio for community-based lung cancer screening overall and according to the three criteria for identifying candidates for screening.
- Screening performance including the following parameters:
    - Cancer detection rate and number needed to screen to detect one lung cancer according to risk criteria over two screening rounds.
    - False positive and false negative rate.
    - Rate of investigation of benign disease and benign surgical resection rate.
    - Attendance by LDCT screening round and according to participant characteristics (age, sex, smoking status, ethnicity and SES).
    - Treatment of screen detected lung cancer including surgical resection rate.
    - Investigations, treatments and adverse events generated from screening including incidental findings.
    - Interval cancers and recall rates in those undergoing screening.
- Smoking prevalence at start and end of the study in intervention arm and usual care arm.
- Participation rates in telephone assessment by age, sex, smoking status, ethnicity and SES.
- Route to diagnosis, histological subtype, stage and treatment of lung cancers in the intervention (LDCT screened group, eligible respondents, non-respondents and ineligible low risk responders) and usual care arms.
- Lung cancer and all-cause mortality by trial arm.
- Numbers of nodules detected, proportion with eventual diagnosis of cancer by: size (volume), Pancan malignancy risk prediction score (36) and volumetry-derived Volume Doubling Time (VDT).
- Prevalence of undiagnosed airflow obstruction and coronary artery calcification in the screened population.
- Quality of life (EQ-5D and SF-12) scores (37, 38).

For peer review only - http://bmjopen.bmj.com/site/about/guidelines.xhtml

Methods

Study design: A two-arm, implementation study using a single-consent Zelen's randomised controlled design (39). Ever smokers, age 55-80, are randomised to either invitation to a telephone assessment followed by a community-based Lung Health Check (LHC) if at high risk (according to any of USPSTF, PLCO, LLP criteria) or usual care (no invitation) (Figure 1).

Setting: YLST is conducted within the catchment area of a single secondary care site - Leeds Teaching Hospitals (LTH). LHCs take place in mobile units at convenient community locations. A single mobile unit (comprising a mobile CT scanner and support accommodation) rotates through community locations across Leeds on a monthly basis.

Participant identification: Potential participants are identified from participating General Practices (GP) based within Leeds Clinical Commissioning Group (CCG). It is anticipated that ≈80 practices will take part. Eligible participants with records indicating they have ever smoked will be identified from groups of GPs on a monthly basis during the two-year recruitment period (November 2018 - October 2020).

Randomisation: To avoid cohabitees being allocated to different arms of the study the unit of randomisation is the household. A 1:1 randomisation programme using simple randomisation without stratification is run monthly to allocate approximately half the households to either the intervention or control arm.

Randomisation cohort eligibility criteria

Inclusion

- Registered with a participating GP

For peer review only - http://bmjopen.bmj.com/site/about/guidelines.xhtml

- Age 55 to 80 years (inclusive) at time of data extraction
- Registered as current or ex-smoker in primary care databases

Exclusion

- Malignant neoplasm of trachea, bronchus, lung, thymus or pleura diagnosed within 5 years
- Any previous diagnosis of metastatic cancer
- On palliative care register (Gold Standards Framework)
- Primary care coded diagnosis of dementia
- Registered type 1 and 2 objection to participation in the GP Extraction Service
- GP coded diagnosis of "severe frailty" or recorded Electronic Frailty Index (eFI) >0.36
- Nursing home residents, or GP coded status of "Housebound"
- CT Thorax within 12 months of data extraction (including high resolution CT thorax, CT thorax (± abdomen/pelvis) with contrast, CT pulmonary angiogram).

Invitation procedure and adherence: Participants in the intervention arm are invited to contact a telephone LHC assessment to check eligibility and to book an appointment. The approach schedule is staggered over a month and includes: a pre-invitation letter, notifying individuals of the LHC service, followed by an invitation letter and up to 2 reminders for non-responders. All letters have the GP digital signature / letterhead. The invitation is accompanied by a low burden information leaflet designed to address psychological barriers to participation. This stepped, low burden and targeted approach was favourably reviewed by the Lung and Mesothelioma Patient Support Group at LTH Trust; it uses material developed in response to research in low SES populations (40) and leaflets used in the Lung Screen Uptake Trial (35).

For peer review only - http://bmjopen.bmj.com/site/about/guidelines.xhtml

Telephone triage: Invitees who contact the telephone triage are assessed for screening eligibility according to USPSTF criteria, and future lung cancer risk using $PLCO_{M2012}$ and LLP (v2) models. Individuals fulfilling at least one of these three criteria are invited to attend a LHC. Participants not eligible for screening receive a '*Keeping your lungs healthy*' leaflet with general advice regarding respiratory health. Current smokers are offered referral to the Leeds Stop Smoking Service.

Lung Health Check: The LHC is a nurse led service. Upon arrival attendees watch a short film describing the LHC process, explaining the context of the study and discussing the benefits and harms of LDCT screening for lung cancer (e.g. overdiagnosis, invasive tests for benign disease, worry about screening results, radiation exposure). A full participant information sheet is also provided. Fully informed written consent for study participation is obtained. LHC components include:

- LHC questionnaire
- Measurement of height and weight, spirometry, oxygen saturation and exhaled carbon monoxide
- Smoking cessation intervention
- Biomarker sub-study
- LDCT scan – undertaken immediately or at a future date, determined by participant preference.

The CT scan is requested by a doctor or research nurse with appropriate Ionising Radiation (Medical Exposure) Regulations (IR(ME)R) accreditation. LHC and screening results are communicated to participants and primary care within four weeks. Participants with obstructive spirometry without a prior diagnosis of Chronic Obstructive Pulmonary Disease (COPD) or asthma are referred to the Community Respiratory Team.

For peer review only - http://bmjopen.bmj.com/site/about/guidelines.xhtml

Smoking cessation provision: Unless explicitly declined, current smokers (smoked within 4 weeks and/or carbon monoxide reading >6 parts/million) see a specialist smoking cessation practitioner (SCP). Support is provided in line with National Institute for Health and Care Excellence Public Health guideline 48 (NICE PH48) and comprises one session of behavioural support and provision of pharmacotherapy at the time of the LHC. Pharmacotherapy may include Nicotine Replacement Therapy (NRT) provided through delegated prescribing, a commercially available e-cigarette, or a varenicline or bupropion prescription to be taken to the participant's GP. Follow up is face-to-face or by telephone, typically weekly for up to 12 weeks. The effectiveness and acceptability of the service, plus that of a personalised intervention is being evaluated in the YESS study, a nested sub-study within YLST (ISRCTN63825779).

Biomarker sub-study: YLST participants are invited to take part in the biomarker sub-study. This is optional and involves blood samples taken at each visit to the support vehicle and in the cancer clinic for those with a positive scan. Samples are transferred to collaborating laboratories (commercial and academic) for analysis. The overarching aims of the biomarker study are to investigate whether biomarkers can improve the efficacy of risk stratified population selection, pulmonary nodule management and screening intensity.

LDCT Screening: YLST participants are offered biennial LDCT screening over two rounds. The baseline round is termed T0 and the second round T2. Pulmonary nodule surveillance may occur 3 months and/or 12 months after baseline - termed T0+3/12 and T0+12/12 respectively. In the second screening rounds these timepoints are T2+3/12 and T2+12/12 (Figure 2).

LDCT Scan: A sixteen channel (or higher) mobile multi-detector CT will be used throughout the study. Participants lie supine with arms above their head and thorax in the midline.

For peer review only - http://bmjopen.bmj.com/site/about/guidelines.xhtml

Imaging is performed during suspended maximal inspiration. No intravenous contrast is administered. The lung parenchyma (lung apices to bases) is scanned in its entirety in a single craniocaudal acquisition. Radiation exposure is minimised whilst maintaining good image quality (effective radiation dose <2mSv). Image reconstruction is standardised and used for any subsequent follow-up examinations. Images are transferred securely from the mobile unit to LTH picture archiving and communication system (PACS).

CT scan reporting: CT scans are reported by a consortium of Consultant Radiologists with a specific interest in thoracic imaging. All radiologists have substantive appointments at National Health Service (NHS) hospitals and contribute regularly to Lung Cancer Multi-Disciplinary Team meetings. Scans are reported using Veolity™ (MeVis), a bespoke software package for lung cancer screening including automated volumetry and computer-aided detection. The same software will be used throughout the study. Reports are categorised as either: negative, indeterminate or positive; each scan may also have an additional 'incidental' finding label. Categories are defined as follows:

- Negative: normal scan, or abnormal scan that does not require further investigation or intervention.
- Indeterminate: indeterminate pulmonary nodule(s) needing surveillance.
- Positive: finding(s) concerning for lung cancer - requires immediate investigation in the Fast Track Lung Cancer Clinic.
- Incidental: other finding(s) that requires clinical review.

Individuals with a negative LDCT scan at T2 are discharged from the study. A false positive is defined as an individual who has been investigated for possible lung cancer in the fast track lung cancer clinic but is eventually found not to have lung cancer.

Quality Assurance: A random selection of negative scans (≈5%) are second read. Any discordance between scan reports is resolved in the screening review meeting.

For peer review only - http://bmjopen.bmj.com/site/about/guidelines.xhtml

Screening review meeting: Comprises a consultant radiologist, consultant respiratory physician and a study nurse or senior clinical trials assistant. Indeterminate, positive and incidental scans are routinely discussed; second read negative CT scans with discordant reports and others flagged for clinical reasons are also discussed.

Pulmonary nodule management algorithms: Nodule management is based on British Thoracic Society guidelines (41), modified for a screening programme. Nodules are categorised solid or sub-solid (SSN). SSN are either pure ground glass (pGGN) or part solid (PSN). Nodules are measured using volumetry. The risk of malignancy is determined using the Brock risk calculator (36). Surveillance for indeterminate pulmonary nodules occurs at 3 and 12 months after detection in most cases, or 12 months from baseline for pGGNs or cysts with discernible walls. Occasionally the surveillance schedule is modified; this will be determined on a case-by-case basis through the screening review meeting.

Statistical Considerations

Power

The primary outcome measure for assessing participation is the proportion of invitees responding to telephone invitation. The anticipated number randomised by household is 62,980 individuals and with 31,490 in the intervention arm this will be reported with high precision. With 14,170 invitees anticipated to undergo assessment by the risk criteria, there is >95% power to detect differences in the proportion being 'risk positive' using pairwise, two-sided McNemar tests at the 5% significance level. Expected values are 24%, 41% and 29% for USPSTF, PLCO and LLP respectively and a correlation of at least 0.3 (based on a survey of ever smokers aged 55-80 conducted in Leeds CCG). Responders are invited to the LHC with LDCT screen if they are positive on at least one of the risk criteria and on this basis, 6,892 are expected to take part in the screening programme.

For peer review only - http://bmjopen.bmj.com/site/about/guidelines.xhtml

The primary outcome measure for USPSTF, PLCO and LLP criteria performance is number of LDCT screen-detected lung cancers; defined as cancers diagnosed following an invited LDCT screen (prevalence or incidence) or follow up of an abnormality detected at these screens. UKLS used the LLP_{v2} risk tool with predicted 5-year risk ≥5% as an inclusion criteria for randomisation to invitation to screen and 2.1% were diagnosed with lung cancer within 12 months (14). Data from NELSON suggests that a similar proportion of cancers are detected at the incidence screening round (42). YLST aims to maximise recruitment of those at high risk (n≈6,892) and we expect to observe 289 screen-detected lung cancers with a detection rate of 4.2% after 4 years. This does not include interval cancers for which there is little prior data but few such cancers are anticipated.

With 289 screen-detected lung cancers there is >95% power to detect differences in the number of cancers detected using pairwise, two-sided McNemar tests for the comparison of USPSTF vs. PLCO and LLP vs. PLCO at the 5% significance level, with expected values of 212 cancers detected by USPSTF (73.5%), 276 cancers detected by PLCO (95.5%) and 225 cancers detected by LLP (78%). The expected correlation among screen-detected cancers is low (0.05 with USPSTF vs. LLP, and 0.1 for other comparisons). These figures are based on a survey of 71 cancers in ever smokers aged 55-80 conducted in LTH (9). Since there is a much smaller difference in the number of cancers detected between the USPSTF and LLP criteria and low correlation, anticipated power to reliably detect a difference is low (25%).

Power to detect differences in advanced lung cancer: The YLST design allows us to evaluate the effect on lung cancer outcomes of (i) invitation to risk assessment followed by screening in ever smokers aged 55-80 (the randomisation cohort) and (ii) LDCT screening in those eligible at risk assessment. The primary outcome for the comparison of targeted community based LDCT screening for lung cancer versus usual care in YLST is incidence of advanced lung cancer

For peer review only - http://bmjopen.bmj.com/site/about/guidelines.xhtml

For peer review only

in the randomisation cohort. Incidence of lung cancer and proportion of advanced disease (stage III or IV) will be reported in the intervention and control groups as a whole, in the expected 17,320 non-respondents, 7,278 ineligible low risk and 6,892 eligible respondents within the intervention group. Breakdown of late stage cancer by non, low and high risk respondents will be used to adjust for observations in the control group and allow evaluation of (ii) using adapted methods for control of non-compliance (43). We will estimate the effect on the distribution of stage after the first four years.

Assuming a lung cancer incidence of 5, 2 and 3 per thousand per year in the eligible, non-eligible and non-respondents, the overall cumulative incidence over four years would be 12.82 per thousand. Due to randomisation we would expect the same in the controls (with slightly higher incidence in the intervention group due to lead time and overdiagnosis). From national rates we would expect 75% of symptomatic cancers to be at stage III or IV compared to 14% in UKLS. Assuming that 25% of all tumours in the screened population (screen detected and symptomatic) are stage III or IV, and this is 75% in non-responders or those at low risk, then the incidence of cancers at stage III or worse in the intervention group would be 7.43 per thousand. Suppose we observe a cumulative four-year incidence of stage III or worse of 9.62 per thousand in the 31,490 in the control group (75% of 12.82), then the power to detect this difference (2-sided testing 5% significance level) is 85%. The Relative Risk (RR) estimate for late-stage cancers would be 0.77 (95%CI 0.60, 0.94), a 23% reduction.

In the LDCT screened group (anticipated n=6,892), we estimate that there will be a cumulative 4-year rate of advanced stage cancers of 0.005 x 4 x 0.25 = 0.005. In the control group, due to randomisation, we infer an identical population of non, non-eligible and eligible potential responders. Assuming that 65% of all cancers in populations unexposed to screening are advanced including in the control group, then in the group of 6,892 potential eligible

For peer review only - http://bmjopen.bmj.com/site/about/guidelines.xhtml

responders in the control arm, we anticipate a cumulative 4-year incidence of advanced cancers of 0.005 x 4 x 0.65 = 0.013. If the latent eligible potential responder group were directly observed, 6,892 subjects per group would confer 99% power to detect the difference in rates of advanced stage disease as significant (5% significance level, 2-sided testing). However, as it is imputed by subtraction of the quantities observed for the unscreened populations in the intervention arm, the standard error of the comparison will be inflated by around 15%, and therefore we will have nearer 85% power. For calculation of estimated outcomes in the latent group and its standard error, see Cuzick *et al* (43).

Analysis

Baseline characteristics by trial arm and a diagram depicting the number and flow of patients through the trial will be presented. Statistical tests will be two-sided using a 5% significance level unless otherwise specified and 95% confidence intervals will be reported as appropriate.

Primary analyses: Descriptive analyses will be used to present the proportion of the population undertaking telephone risk assessment who are screened according to the USPSTF, PLCO and LLP criteria over two rounds of biennial screening. The number of screen-detected lung cancers over 4 years will be compared by risk criteria. Pairwise, two-sided McNemar tests will be used to compare differences between the risk criteria in terms of proportions of cancers detected that were above the risk threshold. Stage distribution and numbers of advanced lung cancers (stage III or IV) will be reported in the intervention and control groups as a whole after the planned two rounds of screening. Cumulative incidence of advanced lung cancer over this period in those allocated to invitation to targeted community based LDCT screening versus those allocated to no invitation will be compared through estimation of the relative risk (RR) and associated 95% confidence intervals using Poisson regression.

For peer review only - http://bmjopen.bmj.com/site/about/guidelines.xhtml

Secondary analyses: Simple descriptive analyses will be used to present data for secondary outcomes including participation rates, characteristics of those attending LDCT screening and adverse events. The number needed to screen (NNS) to detect one lung cancer for each of the three risk criteria over the two rounds of screening will be reported. To calculate NNS, total number of eligible, risk-positive participants attending a LDCT scan on the mobile van will be divided by total number of screen-detected cancers. NNS to detect one lung cancer is the reciprocal of the cancer detection rate. The performance of the risk prediction tools will also be investigated in terms of four-year cumulative incidence of lung cancer for those found eligible and not eligible for LDCT screening. In addition, the performance of the risk models will be assessed for screen-detected early stage cancers.

Health Economic Analysis

A health economic analysis will be conducted with the primary aim of determining the cost-effectiveness of the YLST screening programme. Secondary analysis will explore the expected cost-effectiveness if such an approach were expanded to a national setting, reflecting the worse current lung cancer rates and outcomes in the Yorkshire region, and determine the relative costs and benefits associated with the different screening criteria. Analysis of the uncertainty around the cost-effectiveness estimates will be conducted including scenario and probabilistic sensitivity analysis.

Ethical and regulatory framework

YLST is an implementation study, designed to replicate a possible approach strategy were lung cancer screening to be introduced in the UK. This involves framing the intervention as a 'Lung Health Check' service without specific mention of research in the original approach material. However, the patient population at participating GP surgeries are informed of the research through dissent and opt out posters displayed at the surgery prior to data extraction. This is

For peer review only - http://bmjopen.bmj.com/site/about/guidelines.xhtml

the only contact with a non-invited usual care control population. Those attending a LHC receive a full explanation from the research nurse/senior clinical trial assistant about screening (including the harms and benefits), and the context of LHCs as a health intervention being studied as part of research. Data collection and LDCT screening only proceed following informed consent. In addition, we wish to compare outcomes to the non-invited control group to allow assessment of lung cancer stage and smoking rates between the two arms. With longer follow-up we wish to determine overdiagnosis rates and the effect on lung cancer and all-cause mortality. Participants found to be at lower risk, who do not meet with a research nurse, are sent information about how their data will be used and details of how to opt out. We will track outcomes in three groups who have not consented to study participation: a non-invited usual care control population; invited non-responders and those contacting the telephone triage service but not fulfilling the criteria for LDCT screen. This study design has been approved by the Health Research Authority following review by the Confidentiality Advisory Group (CAG) in order to collect and process this confidential data by a Section 251 exemption. This approach was also reviewed and supported by a patient group recruited through the Yorkshire Cancer Patient Forum.

Trial governance will be supported by a Trial Management Group (TMG), an Independent Data Monitoring Committee (IDMC) and an Independent Trials Steering Committee (TSC). The TMG will oversee the day to day running and progress of the trial. The IDMC is an advisory body to confidentially review interim data and safety and can recommend premature closure or modification of the trial to the TSC. The TSC provides oversight and will consider reports from the TMG and IDMC, as well as external sources. The TSC make the final decision to recommend prematurely closing the trial if considered necessary. The independent members of these committees include experts in the field of cancer screening, respiratory medicine, radiology and statistics, and a patient/public representative sits on the TSC. Findings from the study will

For peer review only - http://bmjopen.bmj.com/site/about/guidelines.xhtml

be written in accordance with the CONSORT Statement (44), submitted for publication to relevant peer-reviewed journals and presented at conferences. A summary of results will be provided for participants on the study website. An independently chaired Biomarker Committee provides oversight to the biomarker sub-study including the assessment of expressions of interest from potential collaborators.

Discussion

YLST will address a number of important issues directly relevant to the implementation of lung cancer screening. The UK does not currently have a national lung cancer screening programme and thus the control group in YLST receive usual care. Despite convincing evidence of mortality reduction in large randomised controlled trials, previous studies have not demonstrated improved lung cancer outcomes across a whole population invited for screening. YLST will assess lung cancer outcomes in the whole intervention population (people undergoing LDCT screening, people at lower risk not eligible for screening, and non-responders) compared to the non-invited control population. YLST will also assess what effect, if any, invitation to LHC has on smoking rates across the intervention and control populations. Previous studies have shown conflicting effects of screening on smoking behaviours (45-47) and typically offered low levels of cessation support. However, such comparisons are subject to bias related to the characteristics of people who are likely to participate in research studies where full consent was required for all participants.

For peer review only

Figure Legends

Figure 1. YLST flow diagram.

Figure 2. Management algorithms for **(A)** solid pulmonary nodules detected in the first screening round (T0); **(B)** solid nodules detected in an incidence round (T0+12/12 or T2) and **(C)** sub-solid nodules detected during screening (pGGN = pure ground glass nodule; PSN = part solid nodule).

For peer review only

For peer review only - http://bmjopen.bmj.com/site/about/guidelines.xhtml

References

1. National Lung Screening Trial Research T, Aberle DR, Adams AM, Berg CD, Black WC, Clapp JD, et al. Reduced lung-cancer mortality with low-dose computed tomographic screening. N Engl J Med. 2011;365(5):395-409.
2. De Koning H, Van Der Aalst C, Ten Haaf K, al. e. Effects of volume CT lung cancer screening: mortality results of the NELSON randomised-controlled population based trial. J Thorac Oncol 2018(IASLC 19th World Conference on Lung Cancer, Toronto, Canada):S185.
3. Pastorino U, Silva M, Sestini S, Sabia F, Boeri M, Cantarutti A, et al. Prolonged lung cancer screening reduced 10-year mortality in the MILD trial: new confirmation of lung cancer screening efficacy. Ann Oncol. 2019.
4. Humphrey LL, Deffebach M, Pappas M, Baumann C, Artis K, Mitchell JP, et al. Screening for lung cancer with low-dose computed tomography: a systematic review to update the US Preventive services task force recommendation. Ann Intern Med. 2013;159(6):411-20.
5. Moyer VA, Force USPST. Screening for lung cancer: U.S. Preventive Services Task Force recommendation statement. Ann Intern Med. 2014;160(5):330-8.
6. Field JK, Devaraj A, Duffy SW, Baldwin DR. CT screening for lung cancer: Is the evidence strong enough? Lung Cancer. 2016;91:29-35.
7. Kovalchik SA, Tammemagi M, Berg CD, Caporaso NE, Riley TL, Korch M, et al. Targeting of low-dose CT screening according to the risk of lung-cancer death. N Engl J Med. 2013;369(3):245-54.
8. Pinsky PF, Berg CD. Applying the National Lung Screening Trial eligibility criteria to the US population: what percent of the population and of incident lung cancers would be covered? J Med Screen. 2012;19(3):154-6.
9. Gracie K, Kennedy MPT, Esterbrook G, Smith G, Blaxill P, Ameri AT, et al. The proportion of lung cancer patients attending UK lung cancer clinics who would have been eligible for low-dose CT screening. Eur Respir J. 2019;54(2).
10. Tammemagi MC, Church TR, Hocking WG, Silvestri GA, Kvale PA, Riley TL, et al. Evaluation of the lung cancer risks at which to screen ever- and never-smokers: screening rules applied to the PLCO and NLST cohorts. PLoS Med. 2014;11(12):e1001764.
11. Ten Haaf K, Bastani M, Cao P, Jeon J, Toumazis I, Han SS, et al. A comparative modeling analysis of risk-based lung cancer screening strategies. J Natl Cancer Inst. 2019.
12. de Koning HJ, Meza R, Plevritis SK, ten Haaf K, Munshi VN, Jeon J, et al. Benefits and harms of computed tomography lung cancer screening strategies: a comparative modeling study for the U.S. Preventive Services Task Force. Ann Intern Med. 2014;160(5):311-20.
13. Tammemagi MC, Katki HA, Hocking WG, Church TR, Caporaso N, Kvale PA, et al. Selection criteria for lung-cancer screening. N Engl J Med. 2013;368(8):728-36.
14. Field JK, Duffy SW, Baldwin DR, Whynes DK, Devaraj A, Brain KE, et al. UK Lung Cancer RCT Pilot Screening Trial: baseline findings from the screening arm provide evidence for the potential implementation of lung cancer screening. Thorax. 2016;71(2):161-70.
15. Iyen-Omofoman B, Hubbard RB, Smith CJ, Sparks E, Bradley E, Bourke A, et al. The distribution of lung cancer across sectors of society in the United Kingdom: a study using national primary care data. BMC Public Health. 2011;11:857.
16. von Wagner C, Baio G, Raine R, Snowball J, Morris S, Atkin W, et al. Inequalities in participation in an organized national colorectal cancer screening programme: results from the first 2.6 million invitations in England. Int J Epidemiol. 2011;40(3):712-8.
17. Sutton S, Wardle J, Taylor T, McCaffery K, Williamson S, Edwards R, et al. Predictors of attendance in the United Kingdom flexible sigmoidoscopy screening trial. J Med Screen. 2000;7(2):99-104.
18. Ford JG, Howerton MW, Lai GY, Gary TL, Bolen S, Gibbons MC, et al. Barriers to recruiting underrepresented populations to cancer clinical trials: a systematic review. Cancer. 2008;112(2):228-42.
19. Gross CP, Filardo G, Mayne ST, Krumholz HM. The impact of socioeconomic status and race on trial participation for older women with breast cancer. Cancer. 2005;103(3):483-91.
20. Sateren WB, Trimble EL, Abrams J, Brawley O, Breen N, Ford L, et al. How sociodemographics, presence of oncology specialists, and hospital cancer programs affect accrual to cancer treatment trials. J Clin Oncol. 2002;20(8):2109-17.

For peer review only - http://bmjopen.bmj.com/site/about/guidelines.xhtml

21. Ali N, Lifford KJ, Carter B, McRonald F, Yadegarfar G, Baldwin DR, et al. Barriers to uptake among high-risk individuals declining participation in lung cancer screening: a mixed methods analysis of the UK Lung Cancer Screening (UKLS) trial. BMJ Open. 2015;5(7):e008254.
22. Balata H, Tonge J, Barber PV, Colligan D, Elton P, Evison M, et al. Attendees of Manchester's Lung Health Check pilot express a preference for community-based lung cancer screening. Thorax. 2019.
23. Reuben DB, Bassett LW, Hirsch SH, Jackson CA, Bastani R. A randomized clinical trial to assess the benefit of offering on-site mobile mammography in addition to health education for older women. AJR Am J Roentgenol. 2002;179(6):1509-14.
24. Cole SR, Young GP, Byrne D, Guy JR, Morcom J. Participation in screening for colorectal cancer based on a faecal occult blood test is improved by endorsement by the primary care practitioner. J Med Screen. 2002;9(4):147-52.
25. Hewitson P, Ward AM, Heneghan C, Halloran SP, Mant D. Primary care endorsement letter and a patient leaflet to improve participation in colorectal cancer screening: results of a factorial randomised trial. Br J Cancer. 2011;105(4):475-80.
26. Cole SR, Smith A, Wilson C, Turnbull D, Esterman A, Young GP. An advance notification letter increases participation in colorectal cancer screening. J Med Screen. 2007;14(2):73-5.
27. Libby G, Bray J, Champion J, Brownlee LA, Birrell J, Gorman DR, et al. Pre-notification increases uptake of colorectal cancer screening in all demographic groups: a randomized controlled trial. J Med Screen. 2011;18(1):24-9.
28. Sabatino SA, Lawrence B, Elder R, Mercer SL, Wilson KM, DeVinney B, et al. Effectiveness of interventions to increase screening for breast, cervical, and colorectal cancers: nine updated systematic reviews for the guide to community preventive services. Am J Prev Med. 2012;43(1):97-118.
29. Everett T, Bryant A, Griffin MF, Martin-Hirsch PP, Forbes CA, Jepson RG. Interventions targeted at women to encourage the uptake of cervical screening. Cochrane Database Syst Rev. 2011(5):CD002834.
30. Quaife SL, Ruparel M, Dickson JL, Beeken RJ, McEwen A, Baldwin DR, et al. Lung Screen Uptake Trial (LSUT): Randomised Controlled Trial Testing Targeted Invitation Materials. Am J Respir Crit Care Med. 2019.
31. Hinde S, Crilly T, Balata H, Bartlett R, Crilly J, Barber P, et al. The cost-effectiveness of the Manchester 'lung health checks', a community-based lung cancer low-dose CT screening pilot. Lung Cancer. 2018;126:119-24.
32. Snowsill T, Yang H, Griffin E, Long L, Varley-Campbell J, Coelho H, et al. Low-dose computed tomography for lung cancer screening in high-risk populations: a systematic review and economic evaluation. Health Technol Assess. 2018;22(69):1-276.
33. Crosbie PA, Balata H, Evison M, Atack M, Bayliss-Brideaux V, Colligan D, et al. Second round results from the Manchester 'Lung Health Check' community-based targeted lung cancer screening pilot. Thorax. 2018.
34. Crosbie PA, Balata H, Evison M, Atack M, Bayliss-Brideaux V, Colligan D, et al. Implementing lung cancer screening: baseline results from a community-based 'Lung Health Check' pilot in deprived areas of Manchester. Thorax. 2019;74(4):405-9.
35. Quaife SL, Ruparel M, Beeken RJ, McEwen A, Isitt J, Nolan G, et al. The Lung Screen Uptake Trial (LSUT): protocol for a randomised controlled demonstration lung cancer screening pilot testing a targeted invitation strategy for high risk and 'hard-to-reach' patients. BMC Cancer. 2016;16(1):281.
36. McWilliams A, Tammemagi MC, Mayo JR, Roberts H, Liu G, Soghrati K, et al. Probability of cancer in pulmonary nodules detected on first screening CT. N Engl J Med. 2013;369(10):910-9.
37. EuroQol--a new facility for the measurement of health-related quality of life. Health Policy. 1990;16(3):199-208.
38. Ware J, Jr., Kosinski M, Keller SD. A 12-Item Short-Form Health Survey: construction of scales and preliminary tests of reliability and validity. Medical care. 1996;34(3):220-33.
39. Zelen M. A new design for randomized clinical trials. N Engl J Med. 1979;300(22):1242-5.
40. Quaife SL, Marlow LAV, McEwen A, Janes SM, Wardle J. Attitudes towards lung cancer screening in socioeconomically deprived and heavy smoking communities: informing screening communication. Health Expect. 2017;20(4):563-73.
41. Callister ME, Baldwin DR, Akram AR, Barnard S, Cane P, Draffan J, et al. British Thoracic Society guidelines for the investigation and management of pulmonary nodules. Thorax. 2015;70 Suppl 2:ii1-ii54.

For peer review only - http://bmjopen.bmj.com/site/about/guidelines.xhtml

42. Horeweg N, van der Aalst CM, Thunnissen E, Nackaerts K, Weenink C, Groen HJ, et al. Characteristics of lung cancers detected by computer tomography screening in the randomized NELSON trial. Am J Respir Crit Care Med. 2013;187(8):848-54.
43. Cuzick J, Edwards R, Segnan N. Adjusting for non-compliance and contamination in randomized clinical trials. Stat Med. 1997;16(9):1017-29.
44. Boutron I, Altman DG, Moher D, Schulz KF, Ravaud P, Group CN. CONSORT Statement for Randomized Trials of Nonpharmacologic Treatments: A 2017 Update and a CONSORT Extension for Nonpharmacologic Trial Abstracts. Ann Intern Med. 2017;167(1):40-7.
45. Brain K, Carter B, Lifford KJ, Burke O, Devaraj A, Baldwin DR, et al. Impact of low-dose CT screening on smoking cessation among high-risk participants in the UK Lung Cancer Screening Trial. Thorax. 2017;72(10):912-8.
46. van der Aalst CM, van den Bergh KA, Willemsen MC, de Koning HJ, van Klaveren RJ. Lung cancer screening and smoking abstinence: 2 year follow-up data from the Dutch-Belgian randomised controlled lung cancer screening trial. Thorax. 2010;65(7):600-5.
47. Ashraf H, Saghir Z, Dirksen A, Pedersen JH, Thomsen LH, Dossing M, et al. Smoking habits in the randomised Danish Lung Cancer Screening Trial with low-dose CT: final results after a 5-year screening programme. Thorax. 2014;69(6):574-9.

For peer review only

For peer review only - http://bmjopen.bmj.com/site/about/guidelines.xhtml

Author Statement: All authors contributed to the development and set up of the study. MEJC and PAJC are responsible for the overall conduct of the study. MEJC, SR, MD, MK, IS are responsible for the day to day running of the study. SR leads the LHC team. MD provides oversight for study thoracic radiology. MEJC, PAJC, RG, DB, HM, PT, SLQ, SH, IS, RDN developed the protocol. RDN, UM provided guidance around the primary care aspects of the study. RLM designed the smoking cessation provision in the study. RB, PAJC contributed the design of the LHC. SLQ, SJ contributed to the patient facing approach literature used. RG, DT developed the study's statistical / analysis plan. SH and MS developed the health economic study design. IS, AC provide project management and NA data management. PAJC, MM, KF are responsible for the conduct of the biomarker study. PAJC, RG, MEJC wrote the manuscript, all authors contributed to, reviewed and approved the final version of the manuscript.

Funding statement: This work was funded by Yorkshire Cancer Research (Award references L403 & L403B).

Acknowledgements: PAJC is supported by the NIHR Manchester Biomedical Research Centre.

Conflicts of Interest: PAJC has received consultation fees and share options from Everest Detection.

PPI/E statement: The study has involved patients / patient advocates throughout its development. This included advising on study design / approach, participant facing literature, the confidential advisory group submission, design and oversight of the biomarker study with membership of the biomarker committee. We will seek advice from patients / public about dissemination of results on the YLST website.

For peer review only - http://bmjopen.bmj.com/site/about/guidelines.xhtml

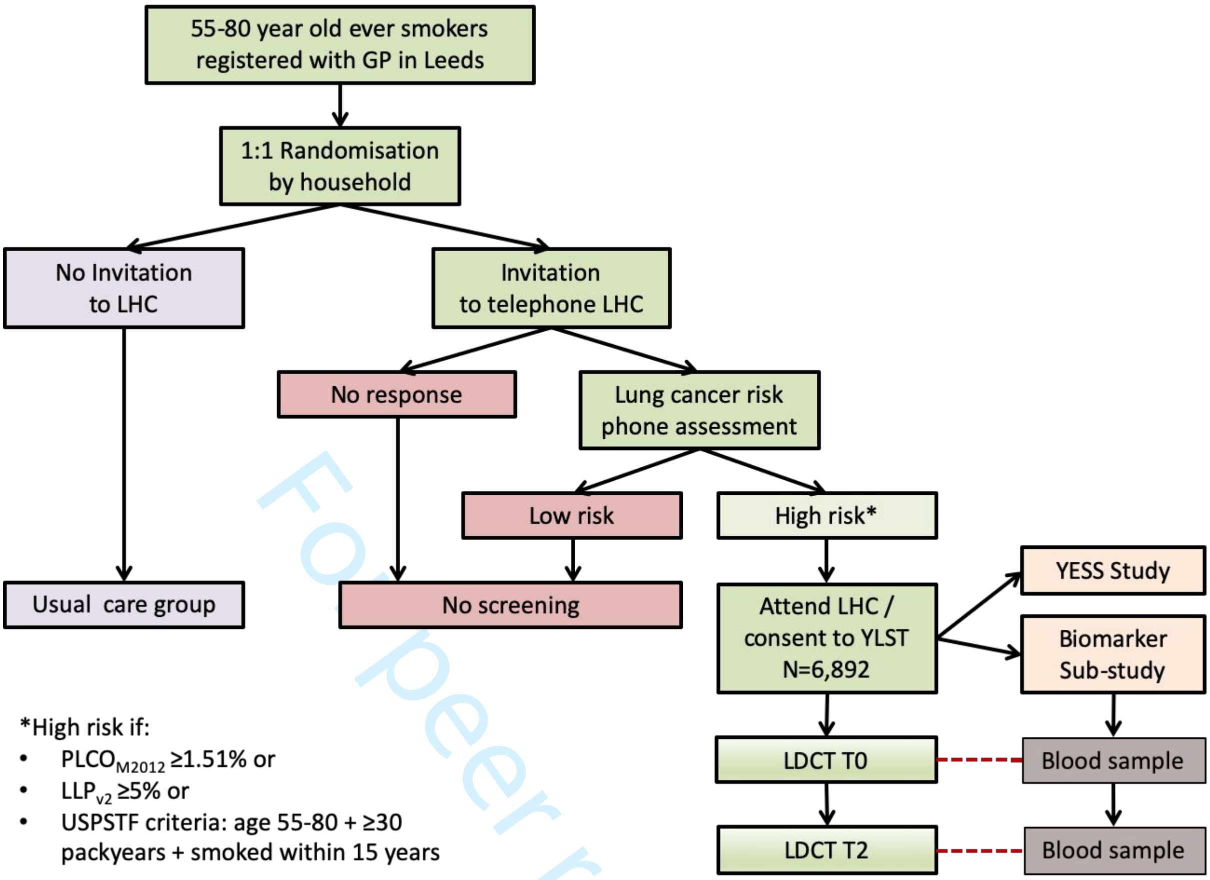

For peer review only

A.

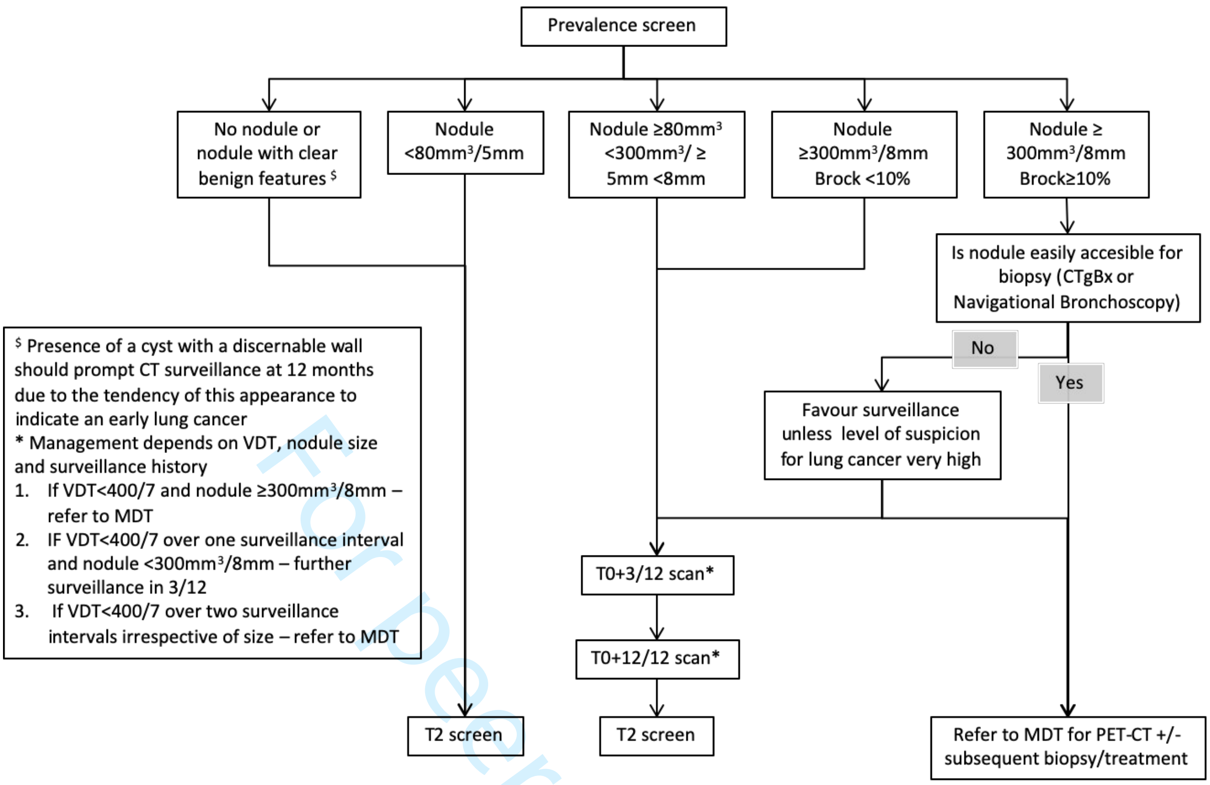

B.

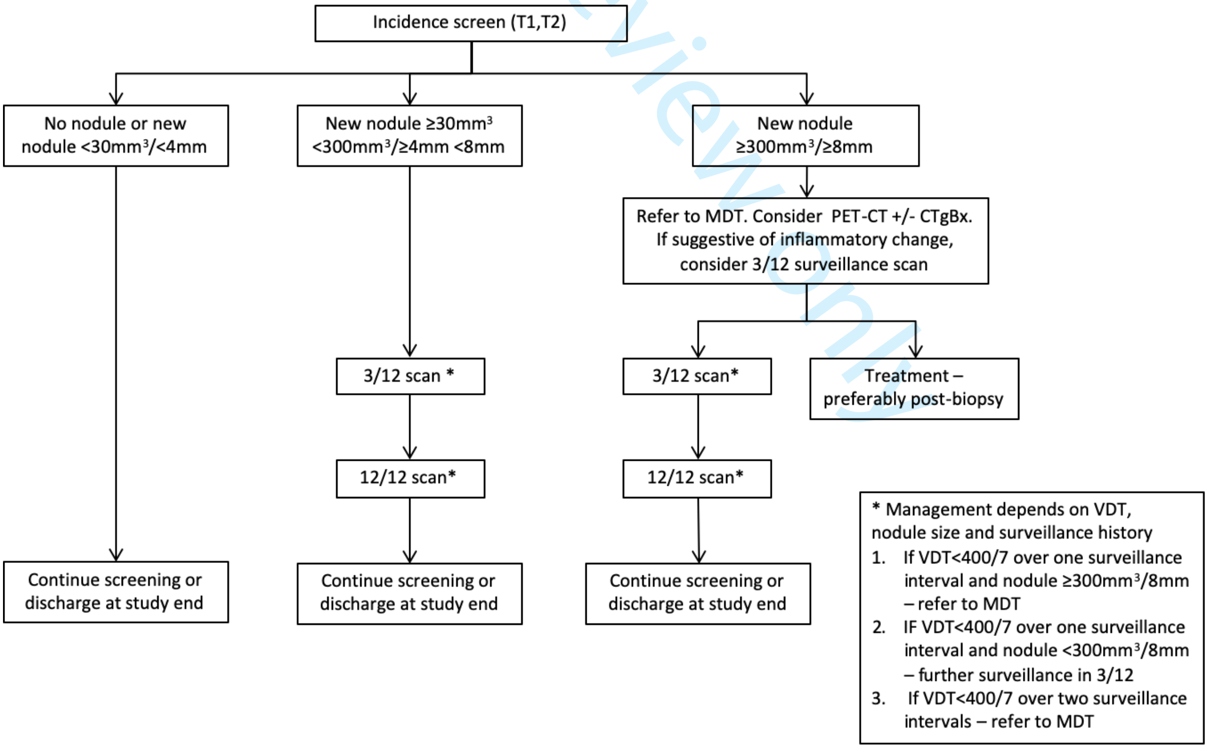

For peer review only

C.

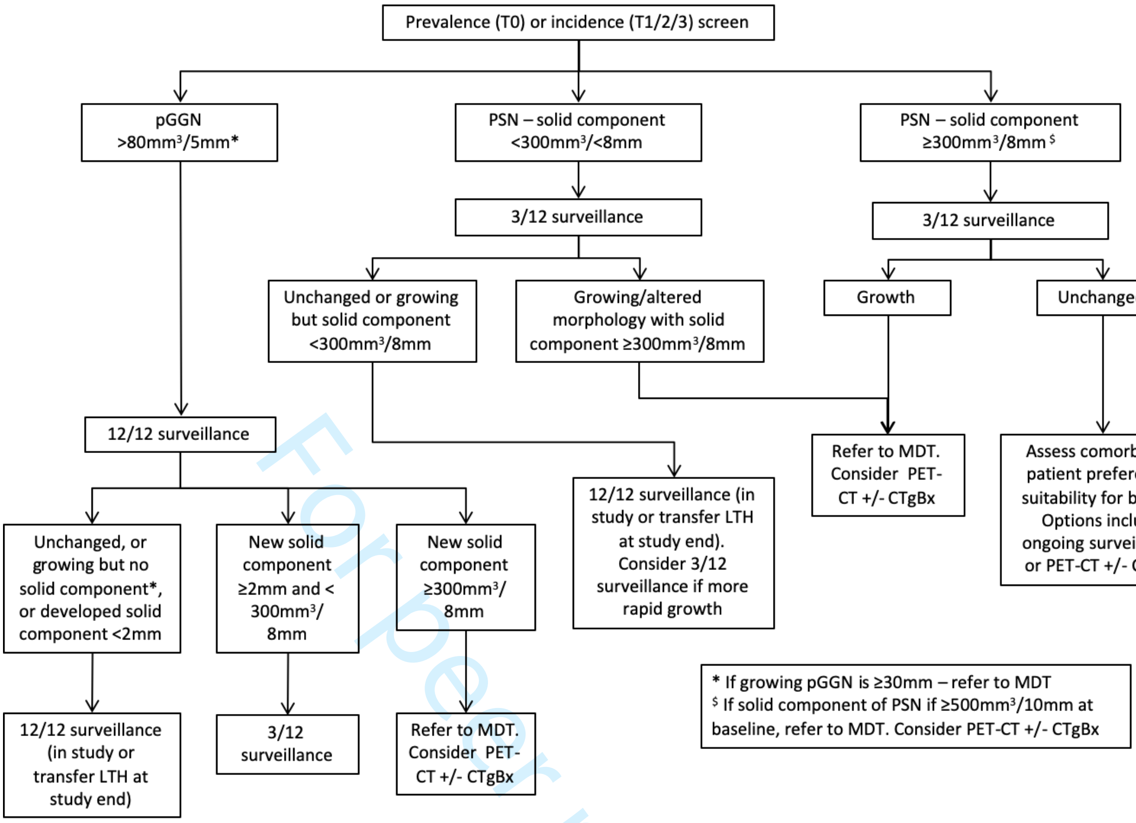

For peer review only

SPIRIT

STANDARD PROTOCOL ITEMS: RECOMMENDATIONS FOR INTERVENTIONAL TRIALS

SPIRIT 2013 Checklist: Recommended items to address in a clinical trial protocol and related documents*

Section/item	Item No	Description
**Administrative information**		
Title	1	Descriptive title identifying the study design, population, interventions, and, if applicable, trial acronym<br>The Yorkshire Lung Screening Trial (YLST): protocol for a randomised controlled trial to evaluate invitation to community-based low dose computed tomography screening for lung cancer versus usual care in a targeted population at risk.
Trial registration	2a	Trial identifier and registry name. If not yet registered, name of intended registry<br>International Standard Registered Clinical Trial Number: ISRCTN42704678
	2b	All items from the World Health Organization Trial Registration Data Set – n/a
Protocol version	3	Date and version identifier – V6.0 19th Nov 2019
Funding	4	Sources and types of financial, material, and other support<br>Funded by Yorkshire Cancer Research (YCR) – further details on page 25 of manuscript.
Roles and responsibilities	5a	Names, affiliations, and roles of protocol contributors<br>Authors and affiliations listed on the title page; contributions detailed on page 25
	5b	Name and contact information for the trial sponsor<br>University of Leeds (UoL) is the trial sponsor.<br>Faculty NHS Research Ethics Officer, Faculty Research Office<br>Level 9, Worsley Building, Clarendon Way, Leeds, LS2 9NL<br>E-mail: governance-ethics@leeds.ac.uk; Tel: 0113 343 7587
	5c	Role of study sponsor and funders, if any, in study design; collection, management, analysis, and interpretation of data; writing of the report; and the decision to submit the report for publication, including whether they will have ultimate authority over any of these activities<br>Neither the funder or sponsor have any role in the analysis / interpretation of data or editorial control over publications.

For peer review only

	5d	Composition, roles, and responsibilities of the coordinating centre, steering committee, endpoint adjudication committee, data management team, and other individuals or groups overseeing the trial, if applicable (see Item 21a for data monitoring committee)<br>The trial's governance / regulatory framework is detailed in the manuscript, pages 19-20.
**Introduction**		
Background and rationale	6a	Description of research question and justification for undertaking the trial, including summary of relevant studies (published and unpublished) examining benefits and harms for each intervention<br>Covered in the introduction pages 4-6
	6b	Explanation for choice of comparators<br>Rationale is detailed on page 6
Objectives	7	Specific objectives or hypotheses<br>Primary and secondary objectives listed on page 7
Trial design	8	Description of trial design including type of trial (eg, parallel group, crossover, factorial, single group), allocation ratio, and framework (eg, superiority, equivalence, noninferiority, exploratory)<br>YLST is a two-arm, implementation study using a single-consent Zelen's randomised controlled design – the design is detailed on page 9.
**Methods: Participants, interventions, and outcomes**		
Study setting	9	Description of study settings (eg, community clinic, academic hospital) and list of countries where data will be collected. Reference to where list of study sites can be obtained<br>YLST is being conducted in the catchment area for a single secondary care site, namely Leeds Teaching Hospitals (LTH). More detail is available in the setting and participants' section (page 9).
Eligibility criteria	10	Inclusion and exclusion criteria for participants. If applicable, eligibility criteria for study centres and individuals who will perform the interventions (eg, surgeons, psychotherapists)<br>Eligibility criteria are detailed on page 10
Interventions	11a	Interventions for each group with sufficient detail to allow replication, including how and when they will be administered<br>The intervention arm receives an invitation to contact a telephone assessment followed by a community-based Lung Health Check (LHC) for those at high risk. The control arm receives no invitation – see page 9. The LHC design and the lung cancer screening programme that follows on from the LHC for those at high risk are detailed on pages 11-13.

For peer review only - http://bmjopen.bmj.com/site/about/guidelines.xhtml

	11b	Criteria for discontinuing or modifying allocated interventions for a given trial participant (eg, drug dose change in response to harms, participant request, or improving/worsening disease)<br>Exclusion criteria are listed on page 10. A participant dissent mechanism is outlined on page 19.
	11c	Strategies to improve adherence to intervention protocols, and any procedures for monitoring adherence (eg, drug tablet return, laboratory tests)<br>The study is based in convenient community locations with free parking to optimise study participation / adherence. We have used a stepped low burden approach to information about study participation (see page 10). Screening adherence is recorded prospectively by the study team.
	11d	Relevant concomitant care and interventions that are permitted or prohibited during the trial<br>There is no change in concomitant care required with trial participants.
Outcomes	12	Primary, secondary, and other outcomes, including the specific measurement variable (eg, systolic blood pressure), analysis metric (eg, change from baseline, final value, time to event), method of aggregation (eg, median, proportion), and time point for each outcome. Explanation of the clinical relevance of chosen efficacy and harm outcomes is strongly recommended<br>Outcome measures are detailed on pages 7-9.
Participant timeline	13	Time schedule of enrolment, interventions (including any run-ins and washouts), assessments, and visits for participants. A schematic diagram is highly recommended (see Figure)<br>The anticipated recruitment period will run from November 2018 to October 2020. The second screening round will then run from November 2020 to October 2022. Surveillance CT scans within the study will continue until October 2023. Figure 1 shows the study schematic.
Sample size	14	Estimated number of participants needed to achieve study objectives and how it was determined, including clinical and statistical assumptions supporting any sample size calculations<br>The justification and rationale for the study size is detailed on pages 14 to 18.
Recruitment	15	Strategies for achieving adequate participant enrolment to reach target sample size<br>The invitational approach is detailed on page 10. The LHC is based in convenient community locations to optimise study accessibility.

Methods: Assignment of interventions (for controlled trials)

Allocation:

Sequence generation	16a	Method of generating the allocation sequence (eg, computer-generated random numbers), and list of any factors for stratification. To reduce predictability of a random sequence, details of any planned restriction (eg, blocking) should be provided in a separate document that is unavailable to those who enrol participants or assign interventions<br>Randomisation takes place after participants (smokers or ex-smokers aged 55-80) have been identified from GP databases and eligibility checks have taken place. The randomisation process is undertaken in batches on a regular basis ahead of invites being sent for the first round of screening (see pages 9-10). The schedule by which General Practices are visited will be determined by practice IMD score, geographical location and proximity to a community screening location. To avoid co-habiting persons being allocated to different arms of the study the unit of randomisation will be the household (see page 9).
Allocation concealment mechanism	16b	Mechanism of implementing the allocation sequence (eg, central telephone; sequentially numbered, opaque, sealed envelopes), describing any steps to conceal the sequence until interventions are assigned<br>A 1:1 randomisation programme using anonymised data and simple randomisation without stratification will be run against the household identifier in order to allocate approximately half the households to the intervention arm and half to the control arm.
Implementation	16c	Who will generate the allocation sequence, who will enrol participants, and who will assign participants to interventions<br>Participants are randomised to receive either an invitation to a LHC or the control arm (no intervention). The trial statistician has oversight of the randomisation / allocation process. Individuals contacting the LHC telephone service will be invited to have a LHC if they meet any one of USPSTF, PLCOM2012 and LLPv2 criteria, see page 11. Recruitment and informed consent is undertaken by a member of the trial team at the time of the LHC.
Blinding (masking)	17a	Who will be blinded after assignment to interventions (eg, trial participants, care providers, outcome assessors, data analysts), and how<br>There is no blinding in the intervention cohort. The control cohort is not approached by the study at any stage.

For peer review only - http://bmjopen.bmj.com/site/about/guidelines.xhtml

	17b	If blinded, circumstances under which unblinding is permissible, and procedure for revealing a participant's allocated intervention during the trial - N/A

Methods: Data collection, management, and analysis

Data collection methods	18a	Plans for assessment and collection of outcome, baseline, and other trial data, including any related processes to promote data quality (eg, duplicate measurements, training of assessors) and a description of study instruments (eg, questionnaires, laboratory tests) along with their reliability and validity, if known. Reference to where data collection forms can be found, if not in the protocol<br>The LHC questionnaire can be found as an appendix of the main protocol – see page 11 of the paper for an outline of data to be collected
	18b	Plans to promote participant retention and complete follow-up, including list of any outcome data to be collected for participants who discontinue or deviate from intervention protocols<br>The LHC is based in convenient locations to optimise accessibility, invitations are followed by reminders for non-responders. Lung cancer outcomes (derived from NCRAS and Leeds Teaching Hospital NLCA submissions), mortality data (ONS) and smoking status (General Practice records) will be collected for all randomised participants, including those who consent to the LHC but later decline biennial LDCT screening at any stage over the two rounds.
Data management	19	Plans for data entry, coding, security, and storage, including any related processes to promote data quality (eg, double data entry; range checks for data values). Reference to where details of data management procedures can be found, if not in the protocol<br>A comprehensive data management plan is detailed in the main protocol (section 7).
Statistical methods	20a	Statistical methods for analysing primary and secondary outcomes. Reference to where other details of the statistical analysis plan can be found, if not in the protocol<br>Primary and secondary analyses are detailed on pages 17-18.
	20b	Methods for any additional analyses (eg, subgroup and adjusted analyses)<br>Health economic analysis is detailed on page 18.
	20c	Definition of analysis population relating to protocol non-adherence (eg, as randomised analysis), and any statistical methods to handle missing data (eg, multiple imputation)<br>See pages 14-18 for an overview of the statistical plan.

Methods: Monitoring

Data monitoring	21a	Composition of data monitoring committee (DMC); summary of its role and reporting structure; statement of whether it is independent from the sponsor and competing interests; and reference to where further details about its charter can be found, if not in the protocol. Alternatively, an explanation of why a DMC is not needed<br>See pages 19-20 for details of the Independent DMC. Charter can be requested from the trial team.
	21b	Description of any interim analyses and stopping guidelines, including who will have access to these interim results and make the final decision to terminate the trial<br>An Independent Data Monitoring Committee (IDMC) will confidentially monitor safety and the accumulating data at least annually. There will be a formal review by the IDMC and Trials Steering Committee (TSC) at one year after the start of LDCT screening with respect to recruitment. Interim analyses will be performed by the trial statisticians. The IDMC will be asked to advise whether the accumulated data from the trial, together with results from other relevant trials, justifies continuing recruitment of further patients or further follow-up. If a decision is made to continue, the IDMC will advise on the frequency of future reviews of the data on the basis of accrual and event rates.
Harms	22	Plans for collecting, assessing, reporting, and managing solicited and spontaneously reported adverse events and other unintended effects of trial interventions or trial conduct<br>AE are not routinely reported as part of the YLST. An SAE will be any untoward medical occurrence that takes place whilst a participant is on the mobile unit and/or which occurs as a result of scanning. SAE will be reported in accordance with Good Clinical Practice and Sponsor requirements (Section 6 of main study protocol).

Auditing	23	Frequency and procedures for auditing trial conduct, if any, and whether the process will be independent from investigators and the sponsor<br>There will be three trial committees: the Trial Management Group (TMG), the IDMC and an independent TSC. The TMG oversee the day to day running of the trial providing clinical, scientific and practical advice and monitoring the progress of the trial regularly. The IDMC is an advisory body to confidentially review interim data and safety and can recommend premature closure or modification of the trial to the TSC. The TSC provides overall supervision and will consider reports from the TMG and IDMC, as well as external sources. The TSC make the final decision to recommend to the funder prematurely closing the trial if considered necessary. The independent members of these committees will include experts in the field of cancer screening, respiratory medicine, radiology and statistics, and a patient/public representative will sit on the TSC. The IDMC and TSC will meet at a frequency as every six month to yearly (see pages 19-20).
**Ethics and dissemination**		
Research ethics approval	24	Plans for seeking research ethics committee/institutional review board (REC/IRB) approval<br>The study has received ethical and CAG approval – page 19.
Protocol amendments	25	Plans for communicating important protocol modifications (eg, changes to eligibility criteria, outcomes, analyses) to relevant parties (eg, investigators, REC/IRBs, trial participants, trial registries, journals, regulators)<br>Any substantial amendments to the study protocol will be submitted to REC and where appropriate to CAG for consideration. The Chief Investigator is responsible for the decision to amend the protocol and will decide whether any amendment is substantial or non-substantial following discussion with the sponsor. A full amendment history will be tracked in the protocol appendix.
Consent or assent	26a	Who will obtain informed consent or assent from potential trial participants or authorised surrogates, and how (see Item 32)<br>This will be undertaken by trained members of the YLST team at the time of the LHC (see page 11).
	26b	Additional consent provisions for collection and use of participant data and biological specimens in ancillary studies, if applicable<br>Participation in the biomarker sub-study requires additional consent – see page 12.

For peer review only - http://bmjopen.bmj.com/site/about/guidelines.xhtml

Confidentiality	27	How personal information about potential and enrolled participants will be collected, shared, and maintained in order to protect confidentiality before, during, and after the trial<br>This is detailed in the data management plan (section 7 of the main study protocol). In summary - data will be stored and analysed in the University of Leeds in a dedicated Virtual Research Environment (VRE) created for the project team. This platform consists of dedicated infrastructure physically and logically separate from other University of Leeds infrastructure.<br><br>The infrastructure comprises dedicated VRE storage and virtual machines with appropriate software, applications and data access set up for each research project. VREs require multi-factor authentication. Following the data gathering phase of the project, data will be pseudonymised using OpenPseudonymiser software, which turns identifiers into a code number with a project-specific key. The code number and key will be securely stored in a different location to the data.
Declaration of interests	28	Financial and other competing interests for principal investigators for the overall trial and each study site<br>Any conflicts of interest or potential conflicts of interest are declared.
Access to data	29	Statement of who will have access to the final trial dataset, and disclosure of contractual agreements that limit such access for investigators<br>Data will be stored securely within the VRE hosted by the sponsor (University of Leeds). Access to the VRE is controlled by a multi-factor authentication and is monitored. Only members of the research team authorised by the chief investigator, on the delegation log, will have access to the dataset. Access will also be granted to those with regulatory oversight responsibilities such as the study Sponsor.<br><br>No personal identifiable data will be shared outside of the research team. Release of anonymised data will be governed by the chief investigator with oversight from the sponsor. Data will only be released from the VRE by the Data Services Team following a check for disclosure which could lead to identification of study participants.

For peer

For peer review only - http://bmjopen.bmj.com/site/about/guidelines.xhtml

Ancillary and post-trial care	30	Provisions, if any, for ancillary and post-trial care, and for compensation to those who suffer harm from trial participation<br>The University of Leeds has sponsor clinical trial insurance. NHS indemnity (for negligent harm) will cover LTH employees, both substantive and honorary, who are working in the course of their NHS employment and in respect of conducting research projects which must have received HRA Permission._Reporting radiologists will have medical indemnity insurance.<br><br>Participants requiring on going medical care related to screening after the trial has stopped will be referred to appropriate local NHS services.
Dissemination policy	31a	Plans for investigators and sponsor to communicate trial results to participants, healthcare professionals, the public, and other relevant groups (eg, via publication, reporting in results databases, or other data sharing arrangements), including any publication restrictions<br>Results will be published in peer reviewed scientific journals and presented at conferences. Results will also be published on the YLST website.
	31b	Authorship eligibility guidelines and any intended use of professional writers<br>Standard definition of author eligibility will be used. There are no plans to use professional writers.
	31c	Plans, if any, for granting public access to the full protocol, participant-level dataset, and statistical code<br>No plans for this
**Appendices**		
Informed consent materials	32	Model consent form and other related documentation given to participants and authorised surrogates<br>Patient facing materials were reviewed and approved by the research ethics committee
Biological specimens	33	Plans for collection, laboratory evaluation, and storage of biological specimens for genetic or molecular analysis in the current trial and for future use in ancillary studies, if applicable<br>There is a separate biomarker study protocol which covers the detail of the biomarker study.

*It is strongly recommended that this checklist be read in conjunction with the SPIRIT 2013 Explanation & Elaboration for important clarification on the items. Amendments to the protocol should be tracked and dated. The SPIRIT checklist is copyrighted by the SPIRIT Group under the Creative Commons "Attribution-NonCommercial-NoDerivs 3.0 Unported" license.

For peer review only - http://bmjopen.bmj.com/site/about/guidelines.xhtml

BMJ Open

The Yorkshire Lung Screening Trial (YLST): protocol for a randomised controlled trial to evaluate invitation to community-based low dose computed tomography screening for lung cancer versus usual care in a targeted population at risk.

Journal:	*BMJ Open*
Manuscript ID	bmjopen-2020-037075.R1
Article Type:	Protocol
Date Submitted by the Author:	24-Jun-2020
Complete List of Authors:	Crosbie, Philip; The University of Manchester, Division of Infection, Immunity and Respiratory Medicine<br>Gabe, Rhian; Queen Mary University of London<br>Simmonds, Irene; University of Leeds<br>Kennedy, Martyn; Leeds Teaching Hospitals NHS Trust,<br>Rogerson, Suzanne; Leeds Teaching Hospitals NHS Trust<br>Ahmed, Nazia; University of Leeds<br>Baldwin, David; Nottingham University Hospitals, City Campus<br>Booton, Richard; Manchester University NHS Foundation Trust<br>Cochrane, Ann; The University of York<br>Darby, Michael; Leeds Teaching Hospitals NHS Trust<br>Franks, Kevin; Leeds Teaching Hospitals NHS Trust, Leeds Cancer Centre, St James's University Hospital<br>Hinde, Sebastian; York University, Centre for Health Economics<br>Janes, Sam; University College London, Respiratory Mediine<br>Macleod, Una; Hull York Medical School<br>Messenger, Mike; University of Leeds<br>Moller, Henrik; Kings College London, Thames Cancer Registry<br>Murray, Rachael; The University of Nottingham, Faculty of Medicine & Health Sciences<br>Neal, Richard; University of Leeds,<br>Quaife, Samantha; University College London, Institutional Research Information Servi<br>Sculpher, Mark; University of York,<br>Tharmanathan, Puvanendran; University of York<br>Torgerson, David; The University of York,<br>CALLISTER, Matthew; Leeds Centre for Respiratory Medicine, St James's University Hospital
**Primary Subject Heading**:	Respiratory medicine
Secondary Subject Heading:	Oncology
Keywords:	Respiratory tract tumours < THORACIC MEDICINE, Respiratory tract tumours < ONCOLOGY, Chest imaging < RADIOLOGY & IMAGING

SCHOLARONE™
Manuscripts

I, the Submitting Author has the right to grant and does grant on behalf of all authors of the Work (as defined in the below author licence), an exclusive licence and/or a non-exclusive licence for contributions from authors who are: i) UK Crown employees; ii) where BMJ has agreed a CC-BY licence shall apply, and/or iii) in accordance with the terms applicable for US Federal Government officers or employees acting as part of their official duties; on a worldwide, perpetual, irrevocable, royalty-free basis to BMJ Publishing Group Ltd ("BMJ") its licensees and where the relevant Journal is co-owned by BMJ to the co-owners of the Journal, to publish the Work in this journal and any other BMJ products and to exploit all rights, as set out in our licence.

The Submitting Author accepts and understands that any supply made under these terms is made by BMJ to the Submitting Author unless you are acting as an employee on behalf of your employer or a postgraduate student of an affiliated institution which is paying any applicable article publishing charge ("APC") for Open Access articles. Where the Submitting Author wishes to make the Work available on an Open Access basis (and intends to pay the relevant APC), the terms of reuse of such Open Access shall be governed by a Creative Commons licence – details of these licences and which Creative Commons licence will apply to this Work are set out in our licence referred to above.

Other than as permitted in any relevant BMJ Author's Self Archiving Policies, I confirm this Work has not been accepted for publication elsewhere, is not being considered for publication elsewhere and does not duplicate material already published. I confirm all authors consent to publication of this Work and authorise the granting of this licence.

For peer review only

The Yorkshire Lung Screening Trial (YLST): protocol for a randomised controlled trial to evaluate invitation to community-based low dose computed tomography screening for lung cancer versus usual care in a targeted population at risk.

Philip AJ Crosbie[1,2], Rhian Gabe[3], Irene Simmonds[4], Martyn Kennedy[5], Suzanne Rogerson[5], Nazia Ahmed[4], David Baldwin[6], Richard Booton[1,2,7], Ann Cochrane[8], Mike Darby[5], Kevin Franks[5], Sebastian Hinde[9], Sam M Janes[10], Una MacLeod[11], Mike Messenger[4], Henrik Møller[12], Rachael L Murray[13], Richard D Neal[4], Samantha L Quaife[14], Mark Sculpher[9], Puvan Tharmanathan[8], David Torgerson[8], Matthew E J Callister[5]*.

[1]Division of Infection, Immunity & Respiratory Medicine, University of Manchester, UK. [2]Manchester Thoracic Oncology Centre, Manchester University NHS Foundation Trust, Manchester, UK. [3]Centre for Cancer Prevention, Queen Mary University of London, UK. [4]Institute of Health Sciences, University of Leeds, UK. [5]Leeds Teaching Hospitals NHS Trust, Leeds, UK. [6]Department of Respiratory Medicine, Nottingham University Hospitals, Nottingham, UK. [7]Manchester Academic Health Science Centre, University of Manchester, UK. [8]York Trials Unit, York, UK. [9]Centre for Health Economics, University of York, UK. [10]Lungs for Living Research Centre, UCL Respiratory, University College London, UK. [11]Hull York Medical School, University of Hull, UK. [12]King's College London, London, UK. [13]Division of Epidemiology and Public Health, University of Nottingham, UK. [14]Research Department of Behavioural Science and Health, University College London, UK.

***Corresponding author:** Dr Mat Callister, St James's University Hospital, Beckett Street, Leeds, UK. LS9 7TF. Tel +44 (0) 113 206 4159. Email: matthew.callister@nhs.net

Word count: 4,718

International Standard Registered Clinical Trial Number: ISRCTN42704678

Keywords: lung cancer screening, lung health check, community, lung cancer risk

For peer review only

Abstract

Introduction: Lung cancer is the world's leading cause of cancer death. Low dose computed tomography (LDCT) screening reduced lung cancer mortality by 20% in the US National Lung Screening Trial (NLST). Here we present the Yorkshire Lung Screening Trial (YLST) which will address key questions of relevance for screening implementation.

Methods and analysis: Using a single-consent Zelen's design, ever-smokers aged 55 to 80 years registered with a GP in Leeds will be randomised (1:1) to invitation to a telephone-based risk-assessment for a Lung Health Check (LHC) or to usual care. The anticipated number randomised by household is 62,980 individuals. Responders at high risk will be invited for LDCT scanning for lung cancer on a mobile van in the community. There will be 2 rounds of screening at an interval of 2 years. Primary objectives are (i) measure participation rates, (ii) compare the performance of $PLCO_{M2012}$ (threshold ≥1.51%), LLP_{v2} (threshold ≥5%) and US Preventive Services Task Force (USPSTF) eligibility criteria for screening population selection, and (iii) assess lung cancer outcomes in the intervention and usual care arms. Secondary evaluations include health economics, quality of life, smoking rates according to intervention arm, screening programme performance with ancillary biomarker, and smoking cessation studies.

Ethics and dissemination: The study has been approved by Greater Manchester West research ethics committee (18-NW-0012) and the Health Research Authority following review by the Confidentiality Advisory Group (CAG). Results will be disseminated through publication in peer-reviewed scientific journals, presentation at conferences and on the YLST website.

Trial registry: ISRCTN42704678 and NCT03750110.

For peer review only - http://bmjopen.bmj.com/site/about/guidelines.xhtml

Strengths and limitations of this study

- Prospective evaluation of three criteria for screening population selection to inform the optimal approach for screening implementation.
- The Zelen's study design and unscreened control group (current standard of care in the UK) will allow us to assess the impact of lung cancer screening at a population level.
- Prospective evaluation of participation rates and clinical outcomes of community-based lung cancer screening with biennial low dose CT scans.
- A nested sub-study within YLST (the Yorkshire Enhanced Stop Smoking (YESS) Trial) will evaluate the effectiveness of a fully integrated co-located stop smoking service in a screening programme.
- The study is not powered to assess the impact of screening on lung cancer specific mortality.

For peer review only

For peer review only - http://bmjopen.bmj.com/site/about/guidelines.xhtml

Introduction

Lung cancer is the leading cause of cancer mortality nationwide, responsible for approximately 30,000 deaths / year in England and Wales. Low dose computed tomography (LDCT) screening reduced mortality in the US National Lung Screening Trial (NLST) which randomised 53,439 participants at high risk to annual LDCT or chest x-ray (CXR) screening for two years (1). LDCT screening reduced lung cancer specific and all-cause mortality by 20% and 6.7% respectively. This finding has recently been confirmed by NELSON trial investigators (2). The MILD study demonstrated a 39% reduction in lung cancer specific mortality over 10 years with LDCT screening (3). In 2014, the US Preventive Services Task Force (USPSTF) recommended LDCT screening for lung cancer based on the NLST findings, extending the upper age to 80 years (4, 5). Despite on-going implementation in the US, several important issues remain unresolved, including optimising identification of high-risk individuals for screening, embedding smoking-cessation in screening programmes, and improving uptake in those at highest risk (6).

Screening selection criteria

Within NLST, very few deaths were prevented in the two lowest lung cancer risk quintiles (7). In addition only 27% of patients diagnosed with lung cancer in the US (8) and 35% in Yorkshire would have been eligible for screening by NLST criteria (9). Composite risk prediction tools show improved sensitivity, specificity and positive predictive value when retrospectively compared to NLST criteria (10). Such tools may therefore identify a greater number of people with lung cancer, and so improve the effectiveness of screening. However, they also tend to identify an older cohort, and thus some of the effect on life years gained and therefore cost-effectiveness may be attenuated (11).

For peer review only - http://bmjopen.bmj.com/site/about/guidelines.xhtml

The Yorkshire Lung Screening Trial (YLST) will prospectively assess three proposed methods of screening population selection: USPSTF, Liverpool Lung Project (LLP), and Prostate, Lung, Colorectal and Ovarian (PLCO) Cancer Screening Trial models. NLST eligibility criteria were age 55-74, ≥30 pack years and smoked within 15 years (1). The USPSTF concluded that screening should be extended to age 80 (12) and issued a recommendation in 2014 (4, 5). The $PLCO_{M2012}$ risk prediction model was derived in 80,375 participants of the PLCO study, and validated in a separate cohort of 37,332 (13). Lung cancer risk ≥1.51% over 6 years was identified as the threshold defining consistent reduced mortality with LDCT screening (10). The UK Lung Cancer Pilot Screening Trial (UKLS) used the LLP model set at a threshold of ≥5% over 5 years to select participants for screening (14). YLST will prospectively assess the performance of USPSTF criteria, the $PLCO_{M2012}$ risk prediction model (6-year risk threshold ≥1.51%) and LLP (version 2) model (5-year risk threshold ≥5%).

Optimising participation for those at most risk

Lung cancer risk is highest within lower socio-economic status (SES) communities (15). Both low SES and current smoking are associated with lower participation in cancer screening programmes (16, 17) and research studies (18-20). In UKLS transport difficulties were the most commonly reported practical barrier to participation (21) especially in lower socioeconomic quintiles, while emotional barriers including higher affective risk perceptions were more frequently reported by smokers. Attendees of Manchester's LHC pilot expressed a preference for community-based screening (22). A randomised trial of mobile mammography for breast screening showed increased participation rates especially in older and low income populations (23). Interventions shown to improve participation in colorectal screening include a primary care endorsement letter, advanced notification of the screening offer and reminder re-invitations (24-28). Enhanced participant information leaflets as well as invitations targeted to address psychological barriers are also promising (29), and in the Lung Screen Uptake Trial

For peer review only - http://bmjopen.bmj.com/site/about/guidelines.xhtml

a low information burden, targeted and stepped invitation approach improved uptake in the lowest SES quintile (30). These factors form part of our invitational strategy.

Cost effectiveness of LDCT screening

LDCT screening needs to ensure the considerable financial investment results in the maximum healthcare gain. Previous UK studies have estimated the incremental cost-effectiveness ratio (ICER) of screening as £8,466 per quality adjusted life year (QALY) in the UKLS (14), £10,069/QALY for mobile LDCT screening in Manchester (31) and £28,169/QALY in a systematic review based analysis (32). The resultant variation in conclusions regarding the cost-effectiveness of screening, driven by different model approaches and populations, highlights the need for further research. By assessing screening yield using different risk tools and thresholds, it will be possible to compare both the clinical and cost effectiveness of different selection criteria.

Rationale

Questions remain about whether the mortality reduction demonstrated in NLST can be translated into routine clinical care. Implementation studies are required to demonstrate participation amongst people at highest risk of lung cancer particularly from deprived populations where the burden of disease is greatest. Basing screening in convenient community locations is one proposed strategy to increase uptake, especially in deprived populations (22). Invitation to a "Lung Health Check" (LHC) not "lung cancer screening" is an approach that has been successfully used to increase participation in previous UK studies leading to screening of higher risk individuals (33-35). Furthermore, clarification of the optimal strategy for defining a high-risk population for screening in the UK would aid any subsequent roll-out of a national screening programme.

For peer review only - http://bmjopen.bmj.com/site/about/guidelines.xhtml

Objectives

The primary objectives of the study are:

1. To measure participation rates of a community-based lung cancer screening programme.
2. To compare the performance of USPSTF, $PLCO_{M2012}$ (≥1.51%) and LLP_{v2} (≥5%) criteria for identifying individuals at high risk of lung cancer for LDCT screening.
3. To assess the clinical outcomes of invitation to targeted community based LDCT screening for lung cancer versus usual care (no invitation).

The study's secondary objectives are:

1. To undertake a health economic evaluation of community based LDCT screening for lung cancer, specifically comparing the health economic impact of the three selection criteria (USPSTF, PLCO, LLP).
2. To evaluate the performance of the screening programme.
3. To determine the effect of invitation to a screening programme on smoking rates comparing intervention and usual care arms.

Outcome measures

The primary outcome measures are:

- The proportion of the study population allocated to intervention who undergo telephone assessment and are screened according to the USPSTF, PLCO and LLP criteria.
- Lung cancers identified in participants selected for LDCT screening according to the USPSTF, PLCO and LLP criteria over two rounds of biennial screening.
- The incidence of advanced lung cancer in the intervention and control arms over the course of the study.

For peer review only - http://bmjopen.bmj.com/site/about/guidelines.xhtml

The secondary outcome measures are:

- Incremental cost-effectiveness ratio for community-based lung cancer screening overall and according to the three criteria for identifying candidates for screening.
- Screening performance including the following parameters:
    - Cancer detection rate and number needed to screen to detect one lung cancer according to risk criteria over two screening rounds.
    - False positive and false negative rate.
    - Rate of investigation of benign disease and benign surgical resection rate.
    - Attendance by LDCT screening round and according to participant characteristics (age, sex, smoking status, ethnicity and SES).
    - Treatment of screen detected lung cancer including surgical resection rate.
    - Investigations, treatments and adverse events generated from screening including incidental findings.
    - Interval cancers and recall rates in those undergoing screening.
- Smoking prevalence at start and end of the study in intervention arm and usual care arm.
- Participation rates in telephone assessment by age, sex, smoking status, ethnicity and SES.
- Route to diagnosis, histological subtype, stage and treatment of lung cancers in the intervention (LDCT screened group, eligible respondents, non-respondents and ineligible low risk responders) and usual care arms.
- Lung cancer and all-cause mortality by trial arm.
- Numbers of nodules detected, proportion with eventual diagnosis of cancer by: size (volume), Pancan malignancy risk prediction score (36) and volumetry-derived Volume Doubling Time (VDT).
- Prevalence of undiagnosed airflow obstruction and coronary artery calcification in the screened population.
- Quality of life (EQ-5D and SF-12) scores (37, 38).

For peer review only - http://bmjopen.bmj.com/site/about/guidelines.xhtml

Methods

Study design: A two-arm, implementation study using a single-consent Zelen's randomised controlled design (39). Ever smokers, age 55-80, are randomised to either invitation to a telephone assessment followed by a community-based Lung Health Check (LHC) if at high risk (according to any of USPSTF, PLCO, LLP criteria) or usual care (no invitation) (Figure 1).

Setting: YLST is conducted within the catchment area of a single secondary care site - Leeds Teaching Hospitals (LTH). LHCs take place in mobile units at convenient community locations. A single mobile unit (comprising a mobile CT scanner and support accommodation) rotates through community locations across Leeds on a monthly basis.

Participant identification: Potential participants are identified from participating General Practices (GP) based within Leeds Clinical Commissioning Group (CCG). It is anticipated that ≈80 practices will take part. Eligible participants with records indicating they have ever smoked will be identified from groups of GPs on a monthly basis during the two-year recruitment period (November 2018 - October 2020).

Randomisation: To avoid cohabitees being allocated to different arms of the study the unit of randomisation is the household. A 1:1 randomisation programme using simple randomisation without stratification is run monthly to allocate approximately half the households to either the intervention or control arm.

Randomisation cohort eligibility criteria

Inclusion

- Registered with a participating GP

For peer review only

For peer review only - http://bmjopen.bmj.com/site/about/guidelines.xhtml

- Age 55 to 80 years (inclusive) at time of data extraction
- Registered as current or ex-smoker in primary care databases

Exclusion

- Malignant neoplasm of trachea, bronchus, lung, thymus or pleura diagnosed within 5 years
- Any previous diagnosis of metastatic cancer
- On palliative care register (Gold Standards Framework)
- Primary care coded diagnosis of dementia
- Registered type 1 and 2 objection to participation in the GP Extraction Service
- GP coded diagnosis of "severe frailty" or recorded Electronic Frailty Index (eFI) >0.36
- Nursing home residents, or GP coded status of "Housebound"
- CT Thorax within 12 months of data extraction (including high resolution CT thorax, CT thorax (± abdomen/pelvis) with contrast, CT pulmonary angiogram).

Invitation procedure and adherence: Participants in the intervention arm are invited to contact a telephone LHC assessment to check eligibility and to book an appointment. The approach schedule is staggered over a month and includes: a pre-invitation letter, notifying individuals of the LHC service, followed by an invitation letter and up to 2 reminders for non-responders. All letters have the GP digital signature / letterhead. The invitation is accompanied by a low burden information leaflet designed to address psychological barriers to participation. This stepped, low burden and targeted approach was favourably reviewed by the Lung and Mesothelioma Patient Support Group at LTH Trust; it uses material developed in response to research in low SES populations (40) and leaflets used in the Lung Screen Uptake Trial (35).

For peer review only - http://bmjopen.bmj.com/site/about/guidelines.xhtml

Telephone triage: Invitees who contact the telephone triage are assessed for screening eligibility according to USPSTF criteria, and future lung cancer risk using $PLCO_{M2012}$ and LLP (v2) models. Individuals fulfilling at least one of these three criteria are invited to attend a LHC. Participants not eligible for screening receive a '*Keeping your lungs healthy*' leaflet with general advice regarding respiratory health. Current smokers are offered referral to the Leeds Stop Smoking Service.

Lung Health Check: The LHC is a nurse led service. Upon arrival attendees watch a short film describing the LHC process, explaining the context of the study and discussing the benefits and harms of LDCT screening for lung cancer (e.g. overdiagnosis, invasive tests for benign disease, worry about screening results, radiation exposure). A full participant information sheet is also provided. Fully informed written consent for study participation is obtained. LHC components include:

- LHC questionnaire
- Measurement of height and weight, spirometry, oxygen saturation and exhaled carbon monoxide
- Smoking cessation intervention
- Biomarker sub-study
- LDCT scan – undertaken immediately or at a future date, determined by participant preference.

The CT scan is requested by a doctor or research nurse with appropriate Ionising Radiation (Medical Exposure) Regulations (IR(ME)R) accreditation. LHC and screening results are communicated to participants and primary care within four weeks. Participants with obstructive spirometry without a prior diagnosis of Chronic Obstructive Pulmonary Disease (COPD) or asthma are referred to the Community Respiratory Team.

For peer review only - http://bmjopen.bmj.com/site/about/guidelines.xhtml

Smoking cessation provision: Unless explicitly declined, current smokers (smoked within 4 weeks and/or carbon monoxide reading >6 parts/million) see a specialist smoking cessation practitioner (SCP). Support is provided in line with National Institute for Health and Care Excellence Public Health guideline 48 (NICE PH48) and comprises one session of behavioural support and provision of pharmacotherapy at the time of the LHC. Pharmacotherapy may include Nicotine Replacement Therapy (NRT) provided through delegated prescribing, a commercially available e-cigarette, or a varenicline or bupropion prescription to be taken to the participant's GP. Follow up is face-to-face or by telephone, typically weekly for up to 12 weeks. The effectiveness and acceptability of the service, plus that of a personalised intervention is being evaluated in the YESS study, a nested sub-study within YLST (ISRCTN63825779) (41).

Biomarker sub-study: YLST participants are invited to take part in the biomarker sub-study. This is optional and involves blood samples taken at each visit to the support vehicle and in the cancer clinic for those with a positive scan. Samples are transferred to collaborating laboratories (commercial and academic) for analysis. The overarching aims of the biomarker study are to investigate whether biomarkers can improve the efficacy of risk stratified population selection, pulmonary nodule management and screening intensity.

LDCT Screening: YLST participants are offered biennial LDCT screening over two rounds. The baseline round is termed T0 and the second round T2. Pulmonary nodule surveillance may occur 3 months and/or 12 months after baseline - termed T0+3/12 and T0+12/12 respectively. In the second screening rounds these timepoints are T2+3/12 and T2+12/12 (Figure 2).

LDCT Scan: A sixteen channel (or higher) mobile multi-detector CT will be used throughout the study. Participants lie supine with arms above their head and thorax in the midline.

For peer review only - http://bmjopen.bmj.com/site/about/guidelines.xhtml

Imaging is performed during suspended maximal inspiration. No intravenous contrast is administered. The lung parenchyma (lung apices to bases) is scanned in its entirety in a single craniocaudal acquisition. Radiation exposure is minimised whilst maintaining good image quality (effective radiation dose <2mSv). Image reconstruction is standardised and used for any subsequent follow-up examinations. Images are transferred securely from the mobile unit to LTH picture archiving and communication system (PACS).

CT scan reporting: CT scans are reported by a consortium of Consultant Radiologists with a specific interest in thoracic imaging. All radiologists have substantive appointments at National Health Service (NHS) hospitals and contribute regularly to Lung Cancer Multi-Disciplinary Team meetings. Scans are reported using Veolity™ (MeVis), a bespoke software package for lung cancer screening including automated volumetry and computer-aided detection. The same software will be used throughout the study. Reports are categorised as either: negative, indeterminate or positive; each scan may also have an additional 'incidental' finding label. Categories are defined as follows:

- Negative: normal scan, or abnormal scan that does not require further investigation or intervention.
- Indeterminate: indeterminate pulmonary nodule(s) needing surveillance.
- Positive: finding(s) concerning for lung cancer - requires immediate investigation in the Fast Track Lung Cancer Clinic.
- Incidental: other finding(s) that requires clinical review.

Individuals with a negative LDCT scan at T2 are discharged from the study. A false positive is defined as an individual who has been investigated for possible lung cancer in the fast track lung cancer clinic but is eventually found not to have lung cancer.

Quality Assurance: A random selection of negative scans (≈5%) are second read. Any discordance between scan reports is resolved in the screening review meeting.

For peer review only - http://bmjopen.bmj.com/site/about/guidelines.xhtml

Screening review meeting: Comprises a consultant radiologist, consultant respiratory physician and a study nurse or senior clinical trials assistant. Indeterminate, positive and incidental scans are routinely discussed; second read negative CT scans with discordant reports and others flagged for clinical reasons are also discussed.

Pulmonary nodule management algorithms: Nodule management is based on British Thoracic Society guidelines (42), modified for a screening programme. Nodules are categorised solid or sub-solid (SSN). SSN are either pure ground glass (pGGN) or part solid (PSN). Nodules are measured using volumetry. The risk of malignancy is determined using the Brock risk calculator (36). Surveillance for indeterminate pulmonary nodules occurs at 3 and 12 months after detection in most cases, or 12 months from baseline for pGGNs or cysts with discernible walls. Occasionally the surveillance schedule is modified; this will be determined on a case-by-case basis through the screening review meeting.

Statistical Considerations

Power

The primary outcome measure for assessing participation is the proportion of invitees responding to telephone invitation. The anticipated number randomised by household is 62,980 individuals and with 31,490 in the intervention arm this will be reported with high precision. With 14,170 invitees anticipated to undergo assessment by the risk criteria, there is >95% power to detect differences in the proportion being 'risk positive' using pairwise, two-sided McNemar tests at the 5% significance level. Expected values are 24%, 41% and 29% for USPSTF, PLCO and LLP respectively and a correlation of at least 0.3 (based on a survey of ever smokers aged 55-80 conducted in Leeds CCG). Responders are invited to the LHC with LDCT screen if they are positive on at least one of the risk criteria and on this basis, 6,892 are expected to take part in the screening programme.

For peer review only - http://bmjopen.bmj.com/site/about/guidelines.xhtml

The primary outcome measure for USPSTF, PLCO and LLP criteria performance is number of LDCT screen-detected lung cancers; defined as cancers diagnosed following an invited LDCT screen (prevalence or incidence) or follow up of an abnormality detected at these screens. UKLS used the LLP_{v2} risk tool with predicted 5-year risk ≥5% as an inclusion criteria for randomisation to invitation to screen and 2.1% were diagnosed with lung cancer within 12 months (14). Data from NELSON suggests that a similar proportion of cancers are detected at the incidence screening round (43). YLST aims to maximise recruitment of those at high risk (n≈6,892) and we expect to observe 289 screen-detected lung cancers with a detection rate of 4.2% after 4 years. This does not include interval cancers for which there is little prior data but few such cancers are anticipated.

With 289 screen-detected lung cancers there is >95% power to detect differences in the number of cancers detected using pairwise, two-sided McNemar tests for the comparison of USPSTF vs. PLCO and LLP vs. PLCO at the 5% significance level, with expected values of 212 cancers detected by USPSTF (73.5%), 276 cancers detected by PLCO (95.5%) and 225 cancers detected by LLP (78%). The expected correlation among screen-detected cancers is low (0.05 with USPSTF vs. LLP, and 0.1 for other comparisons). These figures are based on a survey of 71 cancers in ever smokers aged 55-80 conducted in LTH (9). Since there is a much smaller difference in the number of cancers detected between the USPSTF and LLP criteria and low correlation, anticipated power to reliably detect a difference is low (25%).

Power to detect differences in advanced lung cancer: The YLST design allows us to evaluate the effect on lung cancer outcomes of (i) invitation to risk assessment followed by screening in ever smokers aged 55-80 (the randomisation cohort) and (ii) LDCT screening in those eligible at risk assessment. The primary outcome for the comparison of targeted community based LDCT screening for lung cancer versus usual care in YLST is incidence of advanced lung cancer

For peer review only - http://bmjopen.bmj.com/site/about/guidelines.xhtml

in the randomisation cohort. Incidence of lung cancer and proportion of advanced disease (stage III or IV) will be reported in the intervention and control groups as a whole, in the expected 17,320 non-respondents, 7,278 ineligible low risk and 6,892 eligible respondents within the intervention group. Breakdown of late stage cancer by non, low and high risk respondents will be used to adjust for observations in the control group and allow evaluation of (ii) using adapted methods for control of non-compliance (44). We will estimate the effect on the distribution of stage after the first four years.

Assuming a lung cancer incidence of 5, 2 and 3 per thousand per year in the eligible, non-eligible and non-respondents, the overall cumulative incidence over four years would be 12.82 per thousand. Due to randomisation we would expect the same in the controls (with slightly higher incidence in the intervention group due to lead time and overdiagnosis). From national rates we would expect 75% of symptomatic cancers to be at stage III or IV compared to 14% in UKLS. Assuming that 25% of all tumours in the screened population (screen detected and symptomatic) are stage III or IV, and this is 75% in non-responders or those at low risk, then the incidence of cancers at stage III or worse in the intervention group would be 7.43 per thousand. Suppose we observe a cumulative four-year incidence of stage III or worse of 9.62 per thousand in the 31,490 in the control group (75% of 12.82), then the power to detect this difference (2-sided testing 5% significance level) is 85%. The Relative Risk (RR) estimate for late-stage cancers would be 0.77 (95%CI 0.60, 0.94), a 23% reduction.

In the LDCT screened group (anticipated n=6,892), we estimate that there will be a cumulative 4-year rate of advanced stage cancers of 0.005 x 4 x 0.25 = 0.005. In the control group, due to randomisation, we infer an identical population of non, non-eligible and eligible potential responders. Assuming that 65% of all cancers in populations unexposed to screening are advanced including in the control group, then in the group of 6,892 potential eligible

For peer review only - http://bmjopen.bmj.com/site/about/guidelines.xhtml

responders in the control arm, we anticipate a cumulative 4-year incidence of advanced cancers of 0.005 x 4 x 0.65 = 0.013. If the latent eligible potential responder group were directly observed, 6,892 subjects per group would confer 99% power to detect the difference in rates of advanced stage disease as significant (5% significance level, 2-sided testing). However, as it is imputed by subtraction of the quantities observed for the unscreened populations in the intervention arm, the standard error of the comparison will be inflated by around 15%, and therefore we will have nearer 85% power. For calculation of estimated outcomes in the latent group and its standard error, see Cuzick *et al* (44).

Analysis

Baseline characteristics by trial arm and a diagram depicting the number and flow of patients through the trial will be presented. Statistical tests will be two-sided using a 5% significance level unless otherwise specified and 95% confidence intervals will be reported as appropriate.

Primary analyses: Descriptive analyses will be used to present the proportion of the population undertaking telephone risk assessment who are screened according to the USPSTF, PLCO and LLP criteria over two rounds of biennial screening. The number of screen-detected lung cancers over 4 years will be compared by risk criteria. Pairwise, two-sided McNemar tests will be used to compare differences between the risk criteria in terms of proportions of cancers detected that were above the risk threshold. Stage distribution and numbers of advanced lung cancers (stage III or IV) will be reported in the intervention and control groups as a whole after the planned two rounds of screening. Cumulative incidence of advanced lung cancer over this period in those allocated to invitation to targeted community based LDCT screening versus those allocated to no invitation will be compared through estimation of the relative risk (RR) and associated 95% confidence intervals using Poisson regression.

For peer review only - http://bmjopen.bmj.com/site/about/guidelines.xhtml

Secondary analyses: Simple descriptive analyses will be used to present data for secondary outcomes including participation rates, characteristics of those attending LDCT screening and adverse events. The number needed to screen (NNS) to detect one lung cancer for each of the three risk criteria over the two rounds of screening will be reported. To calculate NNS, total number of eligible, risk-positive participants attending a LDCT scan on the mobile van will be divided by total number of screen-detected cancers. NNS to detect one lung cancer is the reciprocal of the cancer detection rate. The performance of the risk prediction tools will also be investigated in terms of four-year cumulative incidence of lung cancer for those found eligible and not eligible for LDCT screening. In addition, the performance of the risk models will be assessed for screen-detected early stage cancers.

Health Economic Analysis

A health economic analysis will be conducted with the primary aim of determining the cost-effectiveness of the YLST screening programme. Secondary analysis will explore the expected cost-effectiveness if such an approach were expanded to a national setting, reflecting the worse current lung cancer rates and outcomes in the Yorkshire region, and determine the relative costs and benefits associated with the different screening criteria. Analysis of the uncertainty around the cost-effectiveness estimates will be conducted including scenario and probabilistic sensitivity analysis.

Ethics and dissemination

YLST is an implementation study, designed to replicate a possible approach strategy were lung cancer screening to be introduced in the UK. This involves framing the intervention as a 'Lung Health Check' service without specific mention of research in the original approach material. However, the patient population at participating GP surgeries are informed of the research through dissent and opt out posters displayed at the surgery prior to data extraction. This is

For peer review only - http://bmjopen.bmj.com/site/about/guidelines.xhtml

the only contact with a non-invited usual care control population. Those attending a LHC receive a full explanation from the research nurse/senior clinical trial assistant about screening (including the harms and benefits), and the context of LHCs as a health intervention being studied as part of research. Data collection and LDCT screening only proceed following informed consent. In addition, we wish to compare outcomes to the non-invited control group to allow assessment of lung cancer stage and smoking rates between the two arms. With longer follow-up we wish to determine overdiagnosis rates and the effect on lung cancer and all-cause mortality. Participants found to be at lower risk, who do not meet with a research nurse, are sent information about how their data will be used and details of how to opt out. We will track outcomes in three groups who have not consented to study participation: a non-invited usual care control population; invited non-responders and those contacting the telephone triage service but not fulfilling the criteria for LDCT screen. This study design has been approved by the Health Research Authority following review by the Confidentiality Advisory Group (CAG) in order to collect and process this confidential data by a Section 251 exemption. This approach was also reviewed and supported by a patient group recruited through the Yorkshire Cancer Patient Forum.

Trial governance will be supported by a Trial Management Group (TMG), an Independent Data Monitoring Committee (IDMC) and an Independent Trials Steering Committee (TSC). The TMG will oversee the day to day running and progress of the trial. The IDMC is an advisory body to confidentially review interim data and safety and can recommend premature closure or modification of the trial to the TSC. The TSC provides oversight and will consider reports from the TMG and IDMC, as well as external sources. The TSC make the final decision to recommend prematurely closing the trial if considered necessary. The independent members of these committees include experts in the field of cancer screening, respiratory medicine, radiology and statistics, and a patient/public representative sits on the TSC. Findings from the study will

For peer review only - http://bmjopen.bmj.com/site/about/guidelines.xhtml

be written in accordance with the CONSORT Statement (45), submitted for publication to relevant peer-reviewed journals and presented at conferences. A summary of results will be provided for participants on the study website. An independently chaired Biomarker Committee provides oversight to the biomarker sub-study including the assessment of expressions of interest from potential collaborators.

Discussion

YLST will address a number of important issues directly relevant to the implementation of lung cancer screening. The UK does not currently have a national lung cancer screening programme and thus the control group in YLST receive usual care. Despite convincing evidence of mortality reduction in large randomised controlled trials, previous studies have not demonstrated improved lung cancer outcomes across a whole population invited for screening. YLST will assess lung cancer outcomes in the whole intervention population (people undergoing LDCT screening, people at lower risk not eligible for screening, and non-responders) compared to the non-invited control population. YLST will also assess what effect, if any, invitation to LHC has on smoking rates across the intervention and control populations. Previous studies have shown conflicting effects of screening on smoking behaviours (46-48) and typically offered low levels of cessation support. However, such comparisons are subject to bias related to the characteristics of people who are likely to participate in research studies where full consent was required for all participants.

For peer review only - http://bmjopen.bmj.com/site/about/guidelines.xhtml

Figure Legends

Figure 1. YLST flow diagram.

Figure 2. Management algorithms for **(A)** solid pulmonary nodules detected in the first screening round (T0); **(B)** solid nodules detected in an incidence round (T0+12/12 or T2) and **(C)** sub-solid nodules detected during screening (pGGN = pure ground glass nodule; PSN = part solid nodule).

For peer review only

For peer review only - http://bmjopen.bmj.com/site/about/guidelines.xhtml

References

1. National Lung Screening Trial Research T, Aberle DR, Adams AM, Berg CD, Black WC, Clapp JD, et al. Reduced lung-cancer mortality with low-dose computed tomographic screening. N Engl J Med. 2011;365(5):395-409.
2. de Koning HJ, van der Aalst CM, de Jong PA, Scholten ET, Nackaerts K, Heuvelmans MA, et al. Reduced Lung-Cancer Mortality with Volume CT Screening in a Randomized Trial. N Engl J Med. 2020;382(6):503-13.
3. Pastorino U, Silva M, Sestini S, Sabia F, Boeri M, Cantarutti A, et al. Prolonged lung cancer screening reduced 10-year mortality in the MILD trial: new confirmation of lung cancer screening efficacy. Ann Oncol. 2019.
4. Humphrey LL, Deffebach M, Pappas M, Baumann C, Artis K, Mitchell JP, et al. Screening for lung cancer with low-dose computed tomography: a systematic review to update the US Preventive services task force recommendation. Ann Intern Med. 2013;159(6):411-20.
5. Moyer VA, Force USPST. Screening for lung cancer: U.S. Preventive Services Task Force recommendation statement. Ann Intern Med. 2014;160(5):330-8.
6. Field JK, Devaraj A, Duffy SW, Baldwin DR. CT screening for lung cancer: Is the evidence strong enough? Lung Cancer. 2016;91:29-35.
7. Kovalchik SA, Tammemagi M, Berg CD, Caporaso NE, Riley TL, Korch M, et al. Targeting of low-dose CT screening according to the risk of lung-cancer death. N Engl J Med. 2013;369(3):245-54.
8. Pinsky PF, Berg CD. Applying the National Lung Screening Trial eligibility criteria to the US population: what percent of the population and of incident lung cancers would be covered? J Med Screen. 2012;19(3):154-6.
9. Gracie K, Kennedy MPT, Esterbrook G, Smith G, Blaxill P, Ameri AT, et al. The proportion of lung cancer patients attending UK lung cancer clinics who would have been eligible for low-dose CT screening. Eur Respir J. 2019;54(2).
10. Tammemagi MC, Church TR, Hocking WG, Silvestri GA, Kvale PA, Riley TL, et al. Evaluation of the lung cancer risks at which to screen ever- and never-smokers: screening rules applied to the PLCO and NLST cohorts. PLoS Med. 2014;11(12):e1001764.
11. Ten Haaf K, Bastani M, Cao P, Jeon J, Toumazis I, Han SS, et al. A comparative modeling analysis of risk-based lung cancer screening strategies. J Natl Cancer Inst. 2019.
12. de Koning HJ, Meza R, Plevritis SK, ten Haaf K, Munshi VN, Jeon J, et al. Benefits and harms of computed tomography lung cancer screening strategies: a comparative modeling study for the U.S. Preventive Services Task Force. Ann Intern Med. 2014;160(5):311-20.
13. Tammemagi MC, Katki HA, Hocking WG, Church TR, Caporaso N, Kvale PA, et al. Selection criteria for lung-cancer screening. N Engl J Med. 2013;368(8):728-36.
14. Field JK, Duffy SW, Baldwin DR, Whynes DK, Devaraj A, Brain KE, et al. UK Lung Cancer RCT Pilot Screening Trial: baseline findings from the screening arm provide evidence for the potential implementation of lung cancer screening. Thorax. 2016;71(2):161-70.
15. Iyen-Omofoman B, Hubbard RB, Smith CJ, Sparks E, Bradley E, Bourke A, et al. The distribution of lung cancer across sectors of society in the United Kingdom: a study using national primary care data. BMC Public Health. 2011;11:857.
16. von Wagner C, Baio G, Raine R, Snowball J, Morris S, Atkin W, et al. Inequalities in participation in an organized national colorectal cancer screening programme: results from the first 2.6 million invitations in England. Int J Epidemiol. 2011;40(3):712-8.
17. Sutton S, Wardle J, Taylor T, McCaffery K, Williamson S, Edwards R, et al. Predictors of attendance in the United Kingdom flexible sigmoidoscopy screening trial. J Med Screen. 2000;7(2):99-104.
18. Ford JG, Howerton MW, Lai GY, Gary TL, Bolen S, Gibbons MC, et al. Barriers to recruiting underrepresented populations to cancer clinical trials: a systematic review. Cancer. 2008;112(2):228-42.
19. Gross CP, Filardo G, Mayne ST, Krumholz HM. The impact of socioeconomic status and race on trial participation for older women with breast cancer. Cancer. 2005;103(3):483-91.
20. Sateren WB, Trimble EL, Abrams J, Brawley O, Breen N, Ford L, et al. How sociodemographics, presence of oncology specialists, and hospital cancer programs affect accrual to cancer treatment trials. J Clin Oncol. 2002;20(8):2109-17.

For peer review only - http://bmjopen.bmj.com/site/about/guidelines.xhtml

21. Ali N, Lifford KJ, Carter B, McRonald F, Yadegarfar G, Baldwin DR, et al. Barriers to uptake among high-risk individuals declining participation in lung cancer screening: a mixed methods analysis of the UK Lung Cancer Screening (UKLS) trial. BMJ Open. 2015;5(7):e008254.
22. Balata H, Tonge J, Barber PV, Colligan D, Elton P, Evison M, et al. Attendees of Manchester's Lung Health Check pilot express a preference for community-based lung cancer screening. Thorax. 2019.
23. Reuben DB, Bassett LW, Hirsch SH, Jackson CA, Bastani R. A randomized clinical trial to assess the benefit of offering on-site mobile mammography in addition to health education for older women. AJR Am J Roentgenol. 2002;179(6):1509-14.
24. Cole SR, Young GP, Byrne D, Guy JR, Morcom J. Participation in screening for colorectal cancer based on a faecal occult blood test is improved by endorsement by the primary care practitioner. J Med Screen. 2002;9(4):147-52.
25. Hewitson P, Ward AM, Heneghan C, Halloran SP, Mant D. Primary care endorsement letter and a patient leaflet to improve participation in colorectal cancer screening: results of a factorial randomised trial. Br J Cancer. 2011;105(4):475-80.
26. Cole SR, Smith A, Wilson C, Turnbull D, Esterman A, Young GP. An advance notification letter increases participation in colorectal cancer screening. J Med Screen. 2007;14(2):73-5.
27. Libby G, Bray J, Champion J, Brownlee LA, Birrell J, Gorman DR, et al. Pre-notification increases uptake of colorectal cancer screening in all demographic groups: a randomized controlled trial. J Med Screen. 2011;18(1):24-9.
28. Sabatino SA, Lawrence B, Elder R, Mercer SL, Wilson KM, DeVinney B, et al. Effectiveness of interventions to increase screening for breast, cervical, and colorectal cancers: nine updated systematic reviews for the guide to community preventive services. Am J Prev Med. 2012;43(1):97-118.
29. Everett T, Bryant A, Griffin MF, Martin-Hirsch PP, Forbes CA, Jepson RG. Interventions targeted at women to encourage the uptake of cervical screening. Cochrane Database Syst Rev. 2011(5):CD002834.
30. Quaife SL, Ruparel M, Dickson JL, Beeken RJ, McEwen A, Baldwin DR, et al. Lung Screen Uptake Trial (LSUT): Randomised Controlled Trial Testing Targeted Invitation Materials. Am J Respir Crit Care Med. 2019.
31. Hinde S, Crilly T, Balata H, Bartlett R, Crilly J, Barber P, et al. The cost-effectiveness of the Manchester 'lung health checks', a community-based lung cancer low-dose CT screening pilot. Lung Cancer. 2018;126:119-24.
32. Snowsill T, Yang H, Griffin E, Long L, Varley-Campbell J, Coelho H, et al. Low-dose computed tomography for lung cancer screening in high-risk populations: a systematic review and economic evaluation. Health Technol Assess. 2018;22(69):1-276.
33. Crosbie PA, Balata H, Evison M, Atack M, Bayliss-Brideaux V, Colligan D, et al. Second round results from the Manchester 'Lung Health Check' community-based targeted lung cancer screening pilot. Thorax. 2018.
34. Crosbie PA, Balata H, Evison M, Atack M, Bayliss-Brideaux V, Colligan D, et al. Implementing lung cancer screening: baseline results from a community-based 'Lung Health Check' pilot in deprived areas of Manchester. Thorax. 2019;74(4):405-9.
35. Quaife SL, Ruparel M, Beeken RJ, McEwen A, Isitt J, Nolan G, et al. The Lung Screen Uptake Trial (LSUT): protocol for a randomised controlled demonstration lung cancer screening pilot testing a targeted invitation strategy for high risk and 'hard-to-reach' patients. BMC Cancer. 2016;16(1):281.
36. McWilliams A, Tammemagi MC, Mayo JR, Roberts H, Liu G, Soghrati K, et al. Probability of cancer in pulmonary nodules detected on first screening CT. N Engl J Med. 2013;369(10):910-9.
37. EuroQol--a new facility for the measurement of health-related quality of life. Health Policy. 1990;16(3):199-208.
38. Ware J, Jr., Kosinski M, Keller SD. A 12-Item Short-Form Health Survey: construction of scales and preliminary tests of reliability and validity. Medical care. 1996;34(3):220-33.
39. Zelen M. A new design for randomized clinical trials. N Engl J Med. 1979;300(22):1242-5.
40. Quaife SL, Marlow LAV, McEwen A, Janes SM, Wardle J. Attitudes towards lung cancer screening in socioeconomically deprived and heavy smoking communities: informing screening communication. Health Expect. 2017;20(4):563-73.
41. Murray RM, Brain K, Britton J, Quinn-Scoggins HD, Lewis SA, McCutchan G, et al. The Yorkshire Enhanced Stop Smoking study (YESS): a protocol for a randomised controlled trial to evaluate the effect

For peer review only

For peer review only - http://bmjopen.bmj.com/site/about/guidelines.xhtml

of adding a personalised smoking cessation intervention to a lung cancer screening programme. BMJ Open. 2020.
42. Callister ME, Baldwin DR, Akram AR, Barnard S, Cane P, Draffan J, et al. British Thoracic Society guidelines for the investigation and management of pulmonary nodules. Thorax. 2015;70 Suppl 2:ii1-ii54.
43. Horeweg N, van der Aalst CM, Thunnissen E, Nackaerts K, Weenink C, Groen HJ, et al. Characteristics of lung cancers detected by computer tomography screening in the randomized NELSON trial. Am J Respir Crit Care Med. 2013;187(8):848-54.
44. Cuzick J, Edwards R, Segnan N. Adjusting for non-compliance and contamination in randomized clinical trials. Stat Med. 1997;16(9):1017-29.
45. Boutron I, Altman DG, Moher D, Schulz KF, Ravaud P, Group CN. CONSORT Statement for Randomized Trials of Nonpharmacologic Treatments: A 2017 Update and a CONSORT Extension for Nonpharmacologic Trial Abstracts. Ann Intern Med. 2017;167(1):40-7.
46. Brain K, Carter B, Lifford KJ, Burke O, Devaraj A, Baldwin DR, et al. Impact of low-dose CT screening on smoking cessation among high-risk participants in the UK Lung Cancer Screening Trial. Thorax. 2017;72(10):912-8.
47. van der Aalst CM, van den Bergh KA, Willemsen MC, de Koning HJ, van Klaveren RJ. Lung cancer screening and smoking abstinence: 2 year follow-up data from the Dutch-Belgian randomised controlled lung cancer screening trial. Thorax. 2010;65(7):600-5.
48. Ashraf H, Saghir Z, Dirksen A, Pedersen JH, Thomsen LH, Dossing M, et al. Smoking habits in the randomised Danish Lung Cancer Screening Trial with low-dose CT: final results after a 5-year screening programme. Thorax. 2014;69(6):574-9.

For peer review only

For peer review only - http://bmjopen.bmj.com/site/about/guidelines.xhtml

Author Statement: All authors contributed to the development and set up of the study. MEJC and PAJC are responsible for the overall conduct of the study. MEJC, SR, MD, MK, IS are responsible for the day to day running of the study. SR leads the LHC team. MD provides oversight for study thoracic radiology. MEJC, PAJC, RG, DB, HM, PT, SLQ, SH, IS, RDN developed the protocol. RDN, UM provided guidance around the primary care aspects of the study. RLM designed the smoking cessation provision in the study. RB, PAJC contributed the design of the LHC. SLQ, SJ contributed to the patient facing approach literature used. RG, DT developed the study's statistical / analysis plan. SH and MS developed the health economic study design. IS, AC provide project management and NA data management. PAJC, MM, KF are responsible for the conduct of the biomarker study. PAJC, RG, MEJC wrote the manuscript, all authors contributed to, reviewed and approved the final version of the manuscript.

Funding statement: This work was funded by Yorkshire Cancer Research (Award references L403 & L403B).

Acknowledgements: PAJC is supported by the NIHR Manchester Biomedical Research Centre.

Conflicts of Interest: PAJC has received consultation fees and share options from Everest Detection.

PPI/E statement: The study has involved patients / patient advocates throughout its development. This included advising on study design / approach, participant facing literature, the confidential advisory group submission, design and oversight of the biomarker study with membership of the biomarker committee. We will seek advice from patients / public about dissemination of results on the YLST website.

For peer review only

For peer review only - http://bmjopen.bmj.com/site/about/guidelines.xhtml

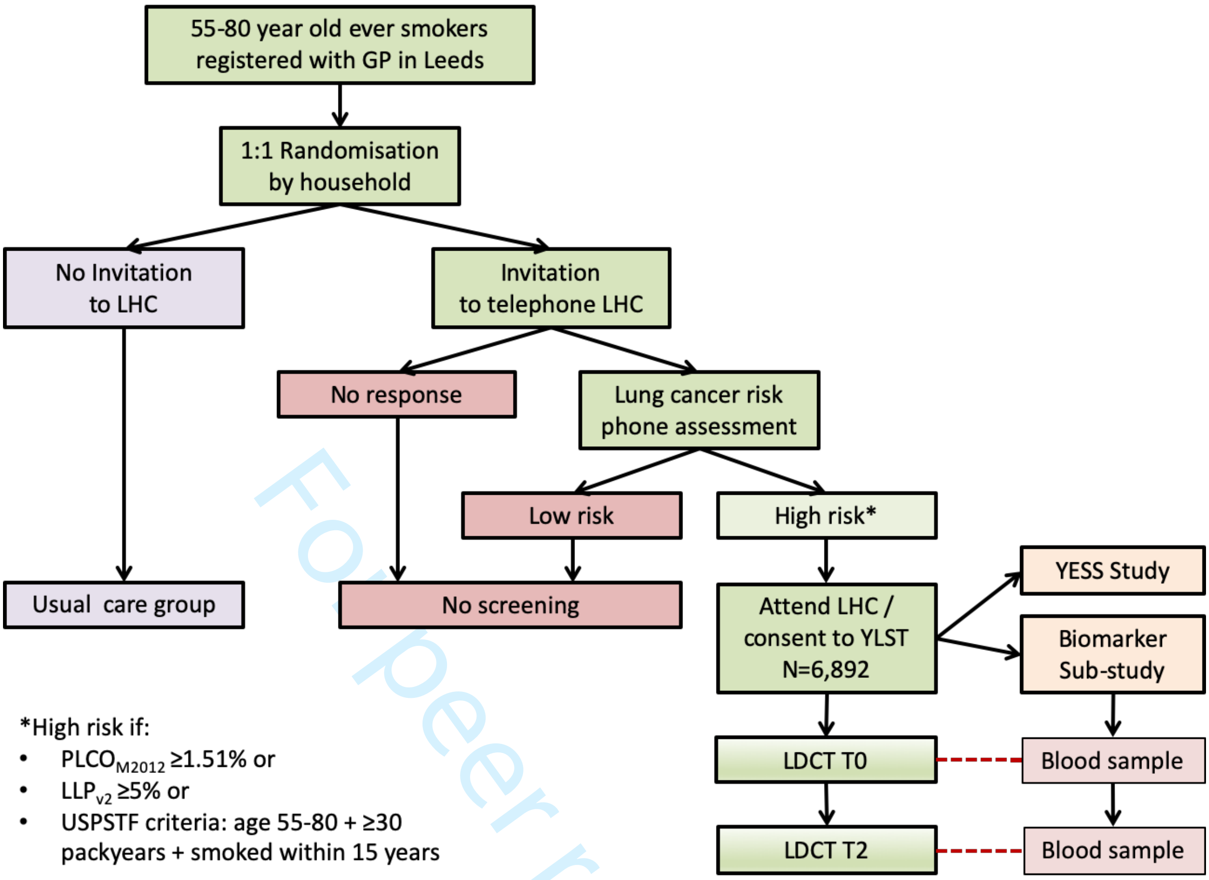

*High risk if:

- $PLCO_{M2012}$ ≥1.51% or
- LLP_{v2} ≥5% or
- USPSTF criteria: age 55-80 + ≥30 packyears + smoked within 15 years

For peer review only

A.

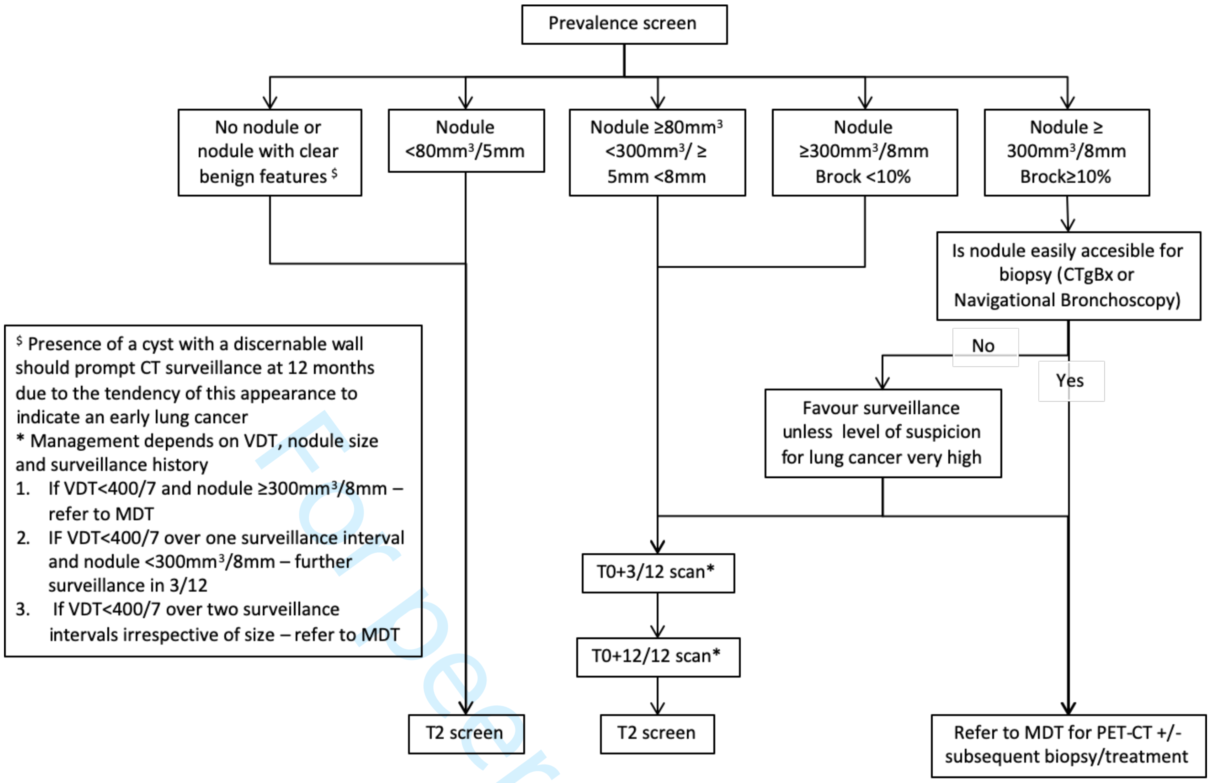

B.

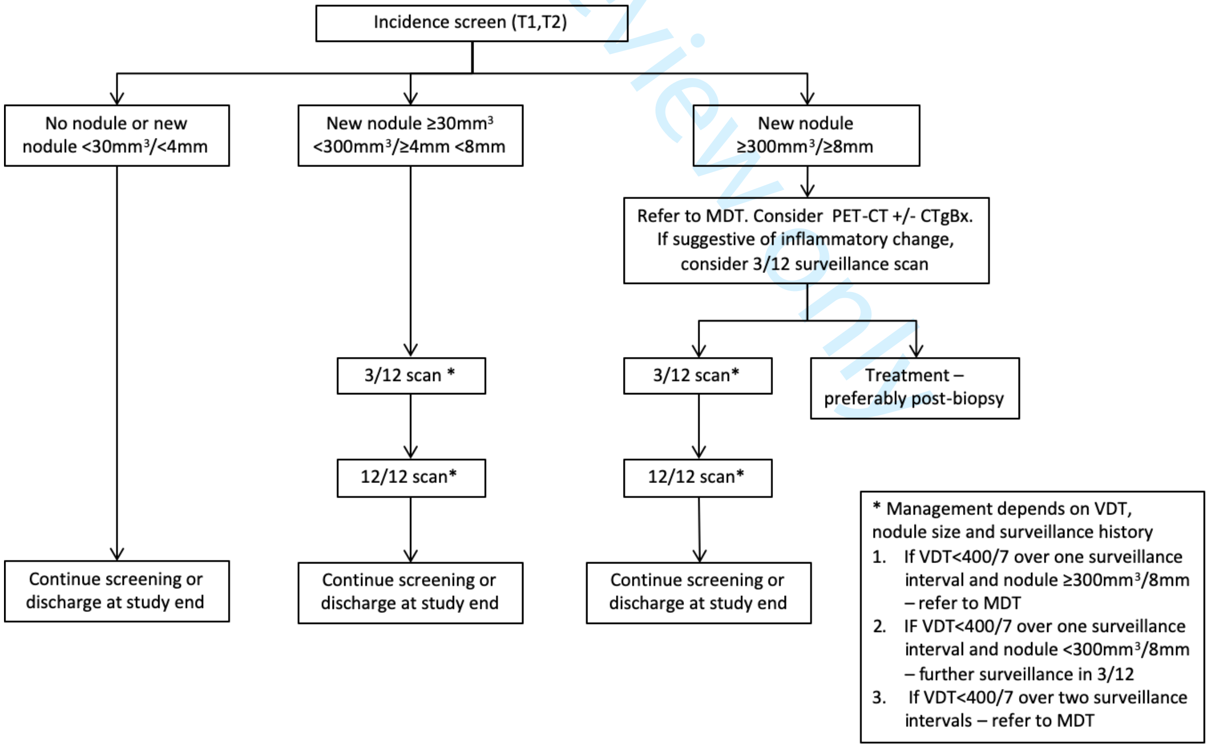

For peer review only

C.

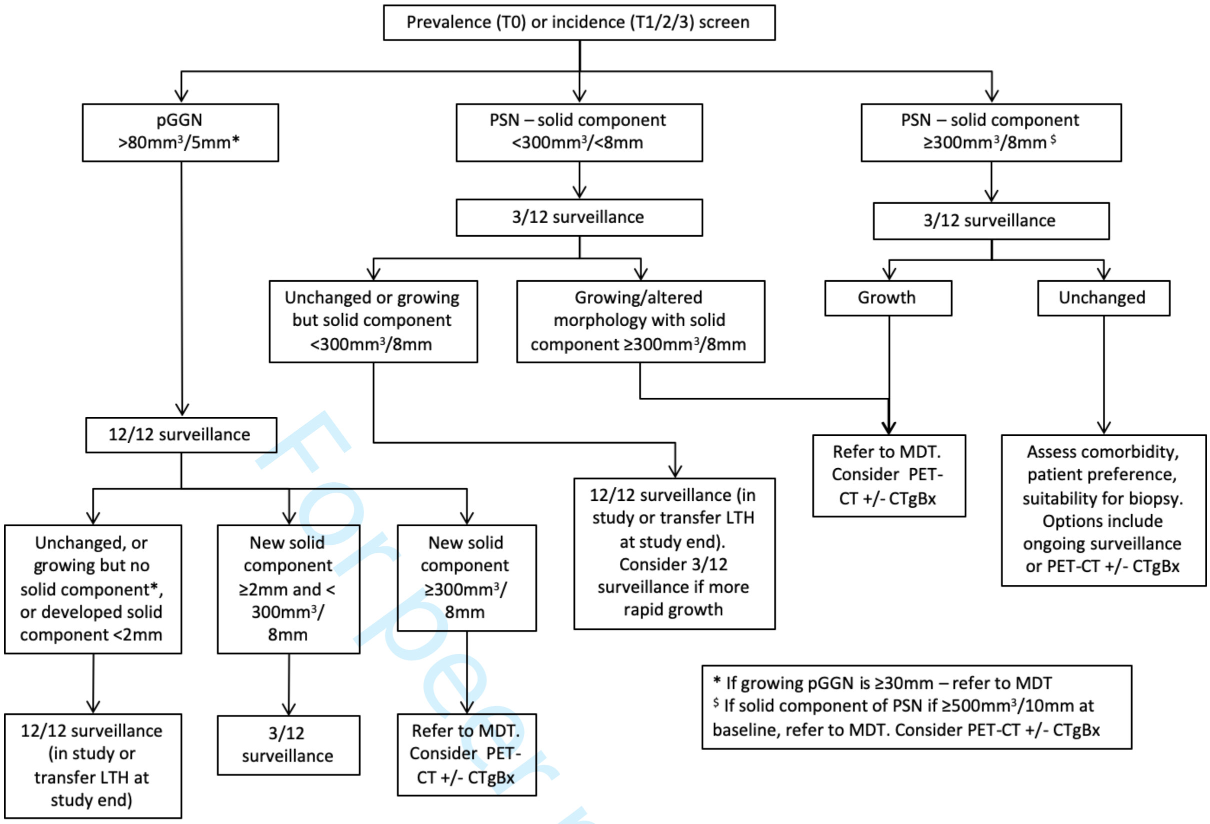

For peer review only

SPIRIT

STANDARD PROTOCOL ITEMS: RECOMMENDATIONS FOR INTERVENTIONAL TRIALS

SPIRIT 2013 Checklist: Recommended items to address in a clinical trial protocol and related documents*

Section/item	Item No	Description
**Administrative information**		
Title	1	Descriptive title identifying the study design, population, interventions, and, if applicable, trial acronym<br>The Yorkshire Lung Screening Trial (YLST): protocol for a randomised controlled trial to evaluate invitation to community-based low dose computed tomography screening for lung cancer versus usual care in a targeted population at risk.
Trial registration	2a	Trial identifier and registry name. If not yet registered, name of intended registry<br>International Standard Registered Clinical Trial Number: ISRCTN42704678
	2b	All items from the World Health Organization Trial Registration Data Set – n/a
Protocol version	3	Date and version identifier – V6.0 19th Nov 2019
Funding	4	Sources and types of financial, material, and other support<br>Funded by Yorkshire Cancer Research (YCR) – further details on page 25 of manuscript.
Roles and responsibilities	5a	Names, affiliations, and roles of protocol contributors<br>Authors and affiliations listed on the title page; contributions detailed on page 25
	5b	Name and contact information for the trial sponsor<br>University of Leeds (UoL) is the trial sponsor.<br>Faculty NHS Research Ethics Officer, Faculty Research Office<br>Level 9, Worsley Building, Clarendon Way, Leeds, LS2 9NL<br>E-mail: governance-ethics@leeds.ac.uk; Tel: 0113 343 7587
	5c	Role of study sponsor and funders, if any, in study design; collection, management, analysis, and interpretation of data; writing of the report; and the decision to submit the report for publication, including whether they will have ultimate authority over any of these activities<br>Neither the funder or sponsor have any role in the analysis / interpretation of data or editorial control over publications.

For peer review only

	5d	Composition, roles, and responsibilities of the coordinating centre, steering committee, endpoint adjudication committee, data management team, and other individuals or groups overseeing the trial, if applicable (see Item 21a for data monitoring committee)<br>The trial's governance / regulatory framework is detailed in the manuscript, pages 19-20.
**Introduction**		
Background and rationale	6a	Description of research question and justification for undertaking the trial, including summary of relevant studies (published and unpublished) examining benefits and harms for each intervention<br>Covered in the introduction pages 4-6
	6b	Explanation for choice of comparators<br>Rationale is detailed on page 6
Objectives	7	Specific objectives or hypotheses<br>Primary and secondary objectives listed on page 7
Trial design	8	Description of trial design including type of trial (eg, parallel group, crossover, factorial, single group), allocation ratio, and framework (eg, superiority, equivalence, noninferiority, exploratory)<br>YLST is a two-arm, implementation study using a single-consent Zelen's randomised controlled design – the design is detailed on page 9.
**Methods: Participants, interventions, and outcomes**		
Study setting	9	Description of study settings (eg, community clinic, academic hospital) and list of countries where data will be collected. Reference to where list of study sites can be obtained<br>YLST is being conducted in the catchment area for a single secondary care site, namely Leeds Teaching Hospitals (LTH). More detail is available in the setting and participants' section (page 9).
Eligibility criteria	10	Inclusion and exclusion criteria for participants. If applicable, eligibility criteria for study centres and individuals who will perform the interventions (eg, surgeons, psychotherapists)<br>Eligibility criteria are detailed on page 10
Interventions	11a	Interventions for each group with sufficient detail to allow replication, including how and when they will be administered<br>The intervention arm receives an invitation to contact a telephone assessment followed by a community-based Lung Health Check (LHC) for those at high risk. The control arm receives no invitation – see page 9. The LHC design and the lung cancer screening programme that follows on from the LHC for those at high risk are detailed on pages 11-13.

For peer review only - http://bmjopen.bmj.com/site/about/guidelines.xhtml

	11b	Criteria for discontinuing or modifying allocated interventions for a given trial participant (eg, drug dose change in response to harms, participant request, or improving/worsening disease)<br>Exclusion criteria are listed on page 10. A participant dissent mechanism is outlined on page 19.
	11c	Strategies to improve adherence to intervention protocols, and any procedures for monitoring adherence (eg, drug tablet return, laboratory tests)<br>The study is based in convenient community locations with free parking to optimise study participation / adherence. We have used a stepped low burden approach to information about study participation (see page 10). Screening adherence is recorded prospectively by the study team.
	11d	Relevant concomitant care and interventions that are permitted or prohibited during the trial<br>There is no change in concomitant care required with trial participants.
Outcomes	12	Primary, secondary, and other outcomes, including the specific measurement variable (eg, systolic blood pressure), analysis metric (eg, change from baseline, final value, time to event), method of aggregation (eg, median, proportion), and time point for each outcome. Explanation of the clinical relevance of chosen efficacy and harm outcomes is strongly recommended<br>Outcome measures are detailed on pages 7-9.
Participant timeline	13	Time schedule of enrolment, interventions (including any run-ins and washouts), assessments, and visits for participants. A schematic diagram is highly recommended (see Figure)<br>The anticipated recruitment period will run from November 2018 to October 2020. The second screening round will then run from November 2020 to October 2022. Surveillance CT scans within the study will continue until October 2023. Figure 1 shows the study schematic.
Sample size	14	Estimated number of participants needed to achieve study objectives and how it was determined, including clinical and statistical assumptions supporting any sample size calculations<br>The justification and rationale for the study size is detailed on pages 14 to 18.
Recruitment	15	Strategies for achieving adequate participant enrolment to reach target sample size<br>The invitational approach is detailed on page 10. The LHC is based in convenient community locations to optimise study accessibility.

For peer review only - http://bmjopen.bmj.com/site/about/guidelines.xhtml

Methods: Assignment of interventions (for controlled trials)

Allocation:

Sequence generation	16a	Method of generating the allocation sequence (eg, computer-generated random numbers), and list of any factors for stratification. To reduce predictability of a random sequence, details of any planned restriction (eg, blocking) should be provided in a separate document that is unavailable to those who enrol participants or assign interventions<br>Randomisation takes place after participants (smokers or ex-smokers aged 55-80) have been identified from GP databases and eligibility checks have taken place. The randomisation process is undertaken in batches on a regular basis ahead of invites being sent for the first round of screening (see pages 9-10). The schedule by which General Practices are visited will be determined by practice IMD score, geographical location and proximity to a community screening location. To avoid co-habiting persons being allocated to different arms of the study the unit of randomisation will be the household (see page 9).
Allocation concealment mechanism	16b	Mechanism of implementing the allocation sequence (eg, central telephone; sequentially numbered, opaque, sealed envelopes), describing any steps to conceal the sequence until interventions are assigned<br>A 1:1 randomisation programme using anonymised data and simple randomisation without stratification will be run against the household identifier in order to allocate approximately half the households to the intervention arm and half to the control arm.
Implementation	16c	Who will generate the allocation sequence, who will enrol participants, and who will assign participants to interventions<br>Participants are randomised to receive either an invitation to a LHC or the control arm (no intervention). The trial statistician has oversight of the randomisation / allocation process. Individuals contacting the LHC telephone service will be invited to have a LHC if they meet any one of USPSTF, PLCOM2012 and LLPv2 criteria, see page 11. Recruitment and informed consent is undertaken by a member of the trial team at the time of the LHC.
Blinding (masking)	17a	Who will be blinded after assignment to interventions (eg, trial participants, care providers, outcome assessors, data analysts), and how<br>There is no blinding in the intervention cohort. The control cohort is not approached by the study at any stage.

For peer review only - http://bmjopen.bmj.com/site/about/guidelines.xhtml

	17b	If blinded, circumstances under which unblinding is permissible, and procedure for revealing a participant's allocated intervention during the trial - N/A

Methods: Data collection, management, and analysis

Data collection methods	18a	Plans for assessment and collection of outcome, baseline, and other trial data, including any related processes to promote data quality (eg, duplicate measurements, training of assessors) and a description of study instruments (eg, questionnaires, laboratory tests) along with their reliability and validity, if known. Reference to where data collection forms can be found, if not in the protocol<br>The LHC questionnaire can be found as an appendix of the main protocol – see page 11 of the paper for an outline of data to be collected
	18b	Plans to promote participant retention and complete follow-up, including list of any outcome data to be collected for participants who discontinue or deviate from intervention protocols<br>The LHC is based in convenient locations to optimise accessibility, invitations are followed by reminders for non-responders. Lung cancer outcomes (derived from NCRAS and Leeds Teaching Hospital NLCA submissions), mortality data (ONS) and smoking status (General Practice records) will be collected for all randomised participants, including those who consent to the LHC but later decline biennial LDCT screening at any stage over the two rounds.
Data management	19	Plans for data entry, coding, security, and storage, including any related processes to promote data quality (eg, double data entry; range checks for data values). Reference to where details of data management procedures can be found, if not in the protocol<br>A comprehensive data management plan is detailed in the main protocol (section 7).
Statistical methods	20a	Statistical methods for analysing primary and secondary outcomes. Reference to where other details of the statistical analysis plan can be found, if not in the protocol<br>Primary and secondary analyses are detailed on pages 17-18.
	20b	Methods for any additional analyses (eg, subgroup and adjusted analyses)<br>Health economic analysis is detailed on page 18.
	20c	Definition of analysis population relating to protocol non-adherence (eg, as randomised analysis), and any statistical methods to handle missing data (eg, multiple imputation)<br>See pages 14-18 for an overview of the statistical plan.

Methods: Monitoring

Data monitoring	21a	Composition of data monitoring committee (DMC); summary of its role and reporting structure; statement of whether it is independent from the sponsor and competing interests; and reference to where further details about its charter can be found, if not in the protocol. Alternatively, an explanation of why a DMC is not needed<br>See pages 19-20 for details of the Independent DMC. Charter can be requested from the trial team.
	21b	Description of any interim analyses and stopping guidelines, including who will have access to these interim results and make the final decision to terminate the trial<br>An Independent Data Monitoring Committee (IDMC) will confidentially monitor safety and the accumulating data at least annually. There will be a formal review by the IDMC and Trials Steering Committee (TSC) at one year after the start of LDCT screening with respect to recruitment. Interim analyses will be performed by the trial statisticians. The IDMC will be asked to advise whether the accumulated data from the trial, together with results from other relevant trials, justifies continuing recruitment of further patients or further follow-up. If a decision is made to continue, the IDMC will advise on the frequency of future reviews of the data on the basis of accrual and event rates.
Harms	22	Plans for collecting, assessing, reporting, and managing solicited and spontaneously reported adverse events and other unintended effects of trial interventions or trial conduct<br>AE are not routinely reported as part of the YLST. An SAE will be any untoward medical occurrence that takes place whilst a participant is on the mobile unit and/or which occurs as a result of scanning. SAE will be reported in accordance with Good Clinical Practice and Sponsor requirements (Section 6 of main study protocol).

Auditing	23	Frequency and procedures for auditing trial conduct, if any, and whether the process will be independent from investigators and the sponsor<br>There will be three trial committees: the Trial Management Group (TMG), the IDMC and an independent TSC. The TMG oversee the day to day running of the trial providing clinical, scientific and practical advice and monitoring the progress of the trial regularly. The IDMC is an advisory body to confidentially review interim data and safety and can recommend premature closure or modification of the trial to the TSC. The TSC provides overall supervision and will consider reports from the TMG and IDMC, as well as external sources. The TSC make the final decision to recommend to the funder prematurely closing the trial if considered necessary. The independent members of these committees will include experts in the field of cancer screening, respiratory medicine, radiology and statistics, and a patient/public representative will sit on the TSC. The IDMC and TSC will meet at a frequency as every six month to yearly (see pages 19-20).
**Ethics and dissemination**		
Research ethics approval	24	Plans for seeking research ethics committee/institutional review board (REC/IRB) approval<br>The study has received ethical and CAG approval – page 19.
Protocol amendments	25	Plans for communicating important protocol modifications (eg, changes to eligibility criteria, outcomes, analyses) to relevant parties (eg, investigators, REC/IRBs, trial participants, trial registries, journals, regulators)<br>Any substantial amendments to the study protocol will be submitted to REC and where appropriate to CAG for consideration. The Chief Investigator is responsible for the decision to amend the protocol and will decide whether any amendment is substantial or non-substantial following discussion with the sponsor. A full amendment history will be tracked in the protocol appendix.
Consent or assent	26a	Who will obtain informed consent or assent from potential trial participants or authorised surrogates, and how (see Item 32)<br>This will be undertaken by trained members of the YLST team at the time of the LHC (see page 11).
	26b	Additional consent provisions for collection and use of participant data and biological specimens in ancillary studies, if applicable<br>Participation in the biomarker sub-study requires additional consent – see page 12.

For peer review only - http://bmjopen.bmj.com/site/about/guidelines.xhtml

Confidentiality	27	How personal information about potential and enrolled participants will be collected, shared, and maintained in order to protect confidentiality before, during, and after the trial<br>This is detailed in the data management plan (section 7 of the main study protocol). In summary - data will be stored and analysed in the University of Leeds in a dedicated Virtual Research Environment (VRE) created for the project team. This platform consists of dedicated infrastructure physically and logically separate from other University of Leeds infrastructure.<br><br>The infrastructure comprises dedicated VRE storage and virtual machines with appropriate software, applications and data access set up for each research project. VREs require multi-factor authentication. Following the data gathering phase of the project, data will be pseudonymised using OpenPseudonymiser software, which turns identifiers into a code number with a project-specific key. The code number and key will be securely stored in a different location to the data.
Declaration of interests	28	Financial and other competing interests for principal investigators for the overall trial and each study site<br>Any conflicts of interest or potential conflicts of interest are declared.
Access to data	29	Statement of who will have access to the final trial dataset, and disclosure of contractual agreements that limit such access for investigators<br>Data will be stored securely within the VRE hosted by the sponsor (University of Leeds). Access to the VRE is controlled by a multi-factor authentication and is monitored. Only members of the research team authorised by the chief investigator, on the delegation log, will have access to the dataset. Access will also be granted to those with regulatory oversight responsibilities such as the study Sponsor.<br><br>No personal identifiable data will be shared outside of the research team. Release of anonymised data will be governed by the chief investigator with oversight from the sponsor. Data will only be released from the VRE by the Data Services Team following a check for disclosure which could lead to identification of study participants.

For peer

Ancillary and post-trial care	30	Provisions, if any, for ancillary and post-trial care, and for compensation to those who suffer harm from trial participation<br>The University of Leeds has sponsor clinical trial insurance. NHS indemnity (for negligent harm) will cover LTH employees, both substantive and honorary, who are working in the course of their NHS employment and in respect of conducting research projects which must have received HRA Permission._Reporting radiologists will have medical indemnity insurance.<br><br>Participants requiring on going medical care related to screening after the trial has stopped will be referred to appropriate local NHS services.
Dissemination policy	31a	Plans for investigators and sponsor to communicate trial results to participants, healthcare professionals, the public, and other relevant groups (eg, via publication, reporting in results databases, or other data sharing arrangements), including any publication restrictions<br>Results will be published in peer reviewed scientific journals and presented at conferences. Results will also be published on the YLST website.
	31b	Authorship eligibility guidelines and any intended use of professional writers<br>Standard definition of author eligibility will be used. There are no plans to use professional writers.
	31c	Plans, if any, for granting public access to the full protocol, participant-level dataset, and statistical code<br>No plans for this
**Appendices**		
Informed consent materials	32	Model consent form and other related documentation given to participants and authorised surrogates<br>Patient facing materials were reviewed and approved by the research ethics committee
Biological specimens	33	Plans for collection, laboratory evaluation, and storage of biological specimens for genetic or molecular analysis in the current trial and for future use in ancillary studies, if applicable<br>There is a separate biomarker study protocol which covers the detail of the biomarker study.

*It is strongly recommended that this checklist be read in conjunction with the SPIRIT 2013 Explanation & Elaboration for important clarification on the items. Amendments to the protocol should be tracked and dated. The SPIRIT checklist is copyrighted by the SPIRIT Group under the Creative Commons "Attribution-NonCommercial-NoDerivs 3.0 Unported" license.

For peer review only - http://bmjopen.bmj.com/site/about/guidelines.xhtml
